# A cohort-based population model for the Poultry Red Mite *Dermanyssus gallinae* (Acari: Dermanyssidae): Application to explore potential pathways for control strategies using Fluralaner as a case study

**DOI:** 10.1371/journal.pone.0357723

**Published:** 2026-10-21

**Authors:** Maroua Bettaieb, Ahmed Louhichi, Mohamed Aziz Darghouth, Slimane Ben Miled

**Affiliations:** 1 École Nationale de Médecine Vétérinaire, Institution de la Recherche et de l’Enseignement Supérieur Agricoles, Université de la Manouba, Sidi Thabet, Tunisia; 2 Laboratoire de bio-(informatique, mathématiques et statistique), Institut Pasteur de Tunis, Université Tunis El-Manar, Tunis, Tunisia; 3 Laboratoire de recherche Ressources Génétiques Animales et Alimentaires (RGAA), Département des Productions Animales, Institut National Agronomiques de Tunisie, Université de Carthage & Institution de la Recherche et de l’Enseignement Supérieur Agricoles, Tunis, Tunisia; 4 École Nationale d’Ingénieurs de Tunis, Université Tunis El-Manar, Tunis, Tunisia; 5 Laboratoire de Recherche Epidemiologie des infections enzootiques des herbivores en Tunisie: application à la lutte (LR02AGR03), Sidi Thabet, Tunisia; Federal University of Espirito Santo, BRAZIL

## Abstract

This project addresses the challenge of controlling *Dermanyssus gallinae* infestations in poultry farms. A cohort-based model is developed that couples the pharmacokinetics of Fluralaner (Exzolt ®) in the plasma of laying hens with the dynamics of the mites, enabling the simulation of various treatment administration strategies while balancing costs. The study reveals that administering a reduced dose of Fluralaner (Exzolt ®) at high frequencies, in conjunction with a customized infestation tolerance threshold for each farm, provides a cost-effective solution for mite control. Specifically, this approach offers significant savings, with up to a 14,15% reduction in treatment costs in a case study involving moderate infestation levels. The findings emphasize that, in many cases, maintaining mite populations below acceptable limits is more economical than complete eradication. This research contributes to advancing poultry farm management practices and optimizing profitability.

## Introduction

The poultry red mite, *Dermanyssus gallinae* (PRM), is a globally significant ectoparasite and a major threat to the economic sustainability and animal welfare standards of industrial poultry production. Its prevalence is particularly acute in laying hen facilities, where extended production cycles -often spanning up to 90 weeks- create an ideal environment for mite populations to establish and proliferate persistently [1]. The cryptic nature of PRM infestations, combined with formidable challenges in achieving eradication, translates into severe economic losses through reduced egg yield and quality, increased mortality, and treatment costs, while simultaneously compromising hen health and welfare [2,3]. Epidemiological studies indicate that approximately 83% of European poultry farms are infested, a statistic reflective of a global problem that spans all production systems [4,5]. The total economic burden in Europe alone is estimated to exceed €150 million annually [2]. Current control paradigms remain heavily reliant on conventional acaricides and inter-flock sanitation. However, these strategies are increasingly recognised as insufficient, often yielding only transient suppression due to the mite’s cryptic behaviour, rapid reproductive potential, and emerging acaricide resistance [6,7]. This failure cycle underscores a pressing need for innovative, knowledge-based management strategies that are both effective and economically optimised. Mathematical modelling offers a powerful tool to meet this need, enabling the simulation of mite population dynamics in response to environmental drivers and control interventions. While such approaches have been successfully applied to other arthropod pests, a comprehensive cohort-based model for *D. gallinae* that integrates pharmacokinetics with population ecology to optimise treatment timing and dosing remains lacking. Therefore, this study aims to develop and validate a robust cohort-based population model for *D. gallinae*. The main objective is to investigate model functions that could be used to optimise control strategies based on acaricidial treatments against *D. gallinae* using a single Fluralaner treatment as a theoretical case study.

## Materials and methods

### Biological and control background

The following sub-section details the key biological and ecological features of *D. gallinae* that form the mechanistic basis for the cohort-based population model developed in this study.

#### Biology and ecology of *Dermanyssus gallinae.*

*Dermanyssus gallinae* is an obligatory but non-host-specific hematophagous mite, capable of feeding on birds and, secondarily, mammals [8]. Its interaction with poultry is transient and nidicolous; mites reside in cracks and crevices within poultry housing, emerging briefly under cover of darkness to feed on resting birds [9]. This cryptic, thigmotactic behaviour, combined with an affinity for environments with 70% + relative humidity and temperatures of 10–35°C, makes colonies difficult to detect and target [10,11]. The reproductive biology of PRM fuels its explosive population growth. After a blood meal, a mated female can lay 1–9 eggs every 12–24 hours, with lifetime fecundity averaging 30 eggs [12]. Egg production increases with successive feedings, peaking after the third to fifth blood meal [13]. The lifecycle, under optimal conditions (28–30°C), can be brief: eggs hatch in 2–3 days into non-feeding larvae, which molt into blood-feeding protonymphs and deutonymphs before reaching adulthood [14]. This rapid turnover enables weekly population doubling in favorable settings [15]. A critical challenge in control is the mite’s remarkable resilience. All mobile stages can survive for extended periods without a host -up to 9 months for nymphs and unfed adults under laboratory conditions- by exploiting microclimates within poultry house refugia [10]. This starvation tolerance, distinct from generational development which requires blood meals, allows populations to persist through sanitary breaks and recolonize rapidly post-treatment.

#### Economic impact and control challenges.

The economic impact of PRM is multifaceted, arising from direct production losses and control costs. Infestations cause anemia, stress, and increased mortality in laying hens, leading directly to reduced egg production and quality issues such as blood spotting [9,16]. Furthermore, PRM acts as a vector for pathogens like *Salmonella* spp., complicating flock health management [17]. Traditional control relies on a limited arsenal of acaricides and physical cleaning. However, these methods are often not sufficiently efficient. The mite’s hidden behaviour protects it from direct contact with most environmental treatments, while resistance to common acaricides is increasingly documented [6]. Sanitary clearance between flocks offers limited long-term benefit, as starvation-resistant nymphs survive to repopulate the house [18]. Consequently, producers often face a costly cycle of temporary suppression and rapid resurgence. However, the development of Fluralaner, marketed in laying hens under the trade name Exzolt ®, has enabled more effective control of the red poultry mite in industrial farming.

#### Rationale for a modelling-based strategy.

The complexity of PRM biology and the suboptimal outcomes of conventional control highlight the need for a more strategic approach. Population models are invaluable tools in IPM, allowing for the in silico testing of intervention strategies against dynamic pest lifecycles. While such models have been developed for ticks and other mites, a comprehensive model for *D. gallinae* that integrates environmental drivers (especially temperature), detailed stage-structured population dynamics, and the pharmacokinetics/pharmacodynamics of modern systemic acaricides like Fluralaner has been offered by Exzolt®, administered via drinking water according to MSD recommendations, presents a promising systemic control option by rendering bird blood lethal to feeding mites. Determining the most cost-effective treatment frequency and intervention thresholds requires understanding the interaction between the decaying drug concentration in hens and the developing mite cohorts seeking a blood meal. A robust model can identify control strategies that maintain mite populations below economically damaging thresholds, a concept often more sustainable and cost-effective than pursuing total eradication. This study addresses this gap by developing a cohort-based model to simulate the optimisation of acaricidial control, using a single Fluralaner treatment as a case study.

### Mathematical formalism of the problem

For breeders and veterinarians, a control strategy is defined by the pair (𝒜,φ) where: 𝒜 represents the administration schedule. The notion of calendar here extends that of frequency by incorporating a certain aperiodicity; and φ represents the Exzolt® dose expressed here in relation to that recommended by the MSD laboratory (i.e.,: φ=1 designates the dose recommended by the laboratory).

#### Defining a control strategy.

Such a definition amounts to searching for the couple (𝒜,φ) that tends to keep the infestation measured in terms of total population per laying hen of *D. gallinae* (all stages combined) below or equal to a certain threshold $\sigma \in [\sigma_{min},\sigma_{max}]$. $\sigma_{min}$ is the threshold at which infestations begin to affect production. $\sigma_{min}$ is estimated at 50 000 individuals per laying hen. $\sigma_{max}$ is the carrying capacity of the population normalized by the number of laying hens on the farm. Choosing such a threshold amounts to expressing the traditional strategy without Exzolt®.

The idea here is to visualise the calendar 𝒜 as a result of choosing the pair (σ,φ), which is then written as 𝒜(σ,φ). Note that the φ dose is the same for all administrations by design. Thus, there are two ways to define a strategy: the first is a static definition based on the (𝒜,φ) pair, which is independent of the *D. gallinae* response but perfectly explicit and understandable by veterinarians; the second is a dynamic definition based on the pair (σ,φ), which is implicit but more useful for our cost modelling. We then propose to create a model that enables us to create a directly executable farm-dependent transition from dynamic to static definition. However, it would also be relevant to allow the model to run in static mode, so as to explore the response of *D. gallinae* population dynamics to certain reference strategies, in particular the traditional one without Exzolt®, where we expect to find classic dynamics comparable to those described [19].

#### Defining the total cost of a control strategy.

We define the cost of a strategy 𝒞(σ,φ) by the decomposition (1). CEx(σ,φ) represents the cost of buying Exzolt® for a strategy defined by a threshold and a dose per laying hen during a breeding cycle; CDg(σ) represents the cost of production losses caused by *D. gallinae* per laying hen during a breeding cycle; and *CC* represents additional costs associated with other control measures.


$$\begin{array}{c} 𝒞(\sigma ,\varphi )=CEx(\sigma ,\varphi )+CDg(\sigma )+CC. \end{array}$$ (1)

The administration strategy is equivalent, in terms of resource consumption, to card(A(σ,φ)) dissolutions of a φ dose of Exzolt® in drinking water. Therefore, the administration costs are reduced to those of purchase. CEx(σ,φ) is thus given by Equation (2) where $\alpha_{CEx}$ is the cost of a single administration at the recommended dose (φ=1). To characterise CDg(σ) on $\left[\sigma_{min},\sigma_{max}\right]$, we decide to model it with an increasing function having a horizontal tangent at $\sigma =\sigma_{min}$. We then choose a parabolic branch via Equation (3) (Table 1). Based on [20], $\alpha_{CEx}$, $\alpha_{CDg}$, and *CC* are given in Table 2. Finding the optimal strategy amounts to solving a total cost optimisation problem on the decision variables $\sigma \in [\sigma_{min},\sigma_{max}]$ and $\varphi \in [\varphi_{min},\varphi_{max}]$ given by Table 2. This dose range has been proven to have no side effects on laying hens or their production. The resulting optimisation problem is written in Equation (4). Unlike the terms CDg(σ) and *CC*, whose definitions are explicit, the term CEx(σ,φ) depends on 𝒜(σ,φ), which is itself implicit. Its estimation will be the main output of the model to be designed.


$$\begin{array}{c} CEx(\sigma ,\varphi )=\alpha_{CEx}\;card(A(\sigma ,\varphi ))\;\varphi . \end{array}$$ (2)

$$\begin{array}{c} CDg(\sigma )=\frac{CDg(\sigma_{max})}{(\sigma_{max}-\sigma_{min}{)}^{2}}(\sigma -\sigma_{min}{)}^{2}. \end{array}$$ (3)

$$\begin{array}{c} \min_{\begin{array}{c} \sigma \in [\sigma_{min},\sigma_{max}]\\ \varphi \in [\varphi_{min},\varphi_{max}] \end{array}}𝒞(\sigma ,\varphi ). \end{array}$$ (4)

**Table 1:** Numerical values of $\alpha_{CEx}$, $CDg(\sigma_{\max})$ and *CC.*

Parameter	Value
$\alpha_{CEx}$	0.13 euro
$CDg(\sigma_{\max})$	0.65 euro
*CC*	0.24 euro

**Table 2:** Optimisation problem bounds.

Parameter	Value
$\sigma_{\min}$	50 000
$\sigma_{\max}$	500 000
$\varphi_{\min}$	0.1
$\varphi_{\max}$	5

### Model overview

Our model simulates the population dynamics of *D. gallinae* in response to different treatment regimes using Exzolt® in a dark building. The model also incorporates the impact of the choice of temperature θ on the growth of *D. gallinae* populations. The final objective is to optimise treatment strategies in terms of dose and timing in order to minimise the impact of these parasites on poultry rearing costs. The model entities generated during the simulation are population cohorts, identified by their date of birth and characterised instantaneously by their population size, their stage of development, and the age of the population at this stage. The development stage is a physiological variable whose value can take the following forms: egg (*E*), larva (*L*), protonymph (*P*), deutonymph (*D*), pre-reproductive adult (*A*_1_), and reproductive adult (*A*_2_).

Assuming that the individuals in each developmental stage at initialization are the same age, we can visualise initialization as the superposition of six cohorts, one for each developmental stage. We refer to them as “Cohort (*E*)”, “Cohort (*L*)”, “Cohort (*P*)”, “Cohort (*D*)”, “Cohort (*A*_1_)”, and “Cohort (*A*_2_)”. This naming convention addresses the impossibility of identifying these cohorts with positive birth dates at time *t*. However, the characterization remains valid.

Our structuring provides a compact representation of three classical structures. First, it involves structuring by cohort, which groups together individuals exposed to the same environment during their development. Second, structuring by development stage within the same cohort offers a twofold advantage: from a dynamic perspective, it highlights the physiological differences between the various stages, and it also allows us to explicitly show the dates of blood meals associated with each transtadial passage, along with the resulting mortality probabilities. Finally, age-structured breeding adults from different cohorts are considered to rigorously characterise their maternity and instantaneous mortality risk. The Fig 1 provides an exploded view of the dynamics of a single typical cohort.

**Fig 1:**
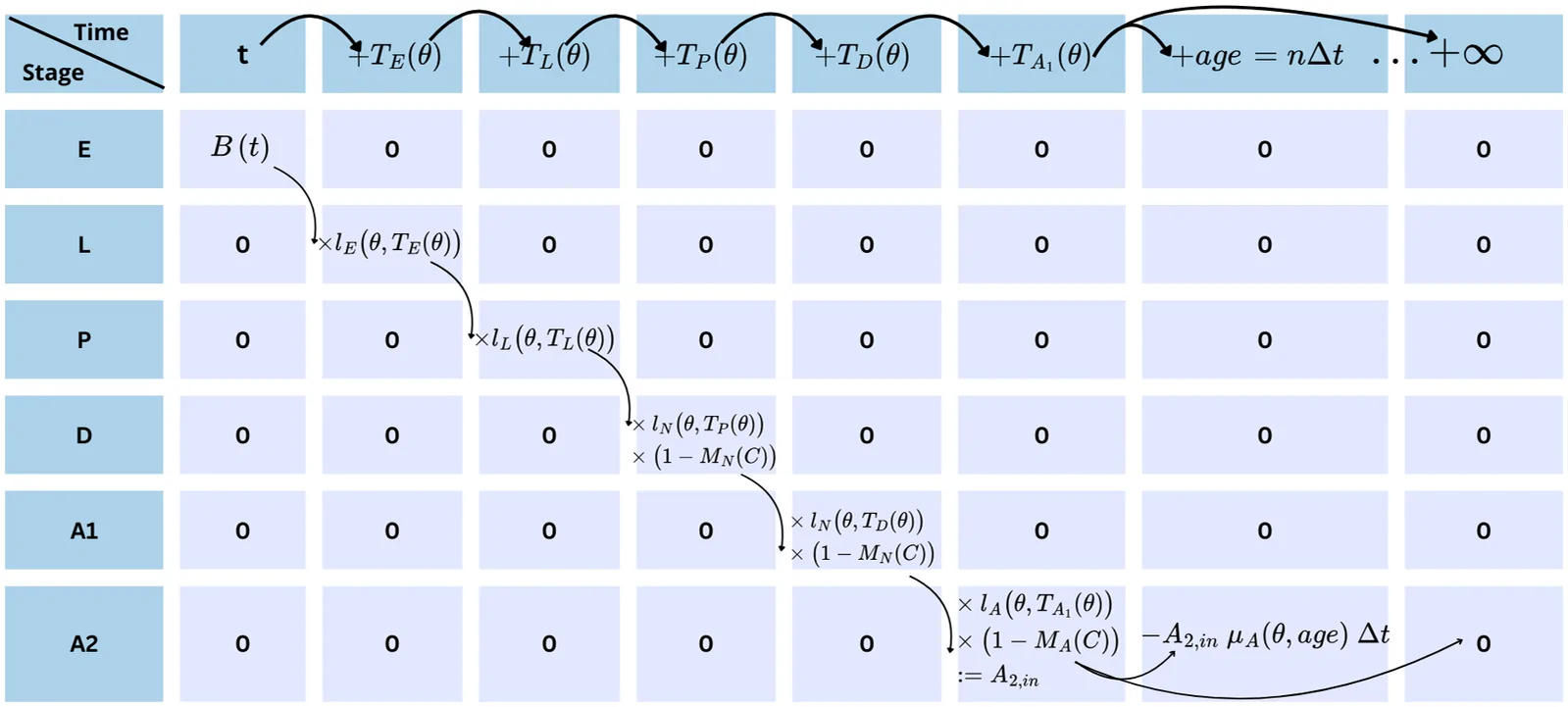
Vector representation of the dynamics of a cohort born at a time *t* > 0.

At time *t*, a cohort is created according to a birth or renewal term *B*(*t*) at stage (*E*). After a time $T_{E}(\theta )$, a fraction of the population passes to the larval stage according to a longevity term $l_{E}(\theta ,T_{E}(\theta ))$. In the same way, after an additional time $T_{L}(\theta )$, only a fraction of the population passes to the (*P*) stage according to a longevity term $l_{L}(\theta ,T_{L}(\theta ))$. The (*P*) stage is the first stage that requires a blood meal. This results in a natural composite longevity $l_{P}(\theta ,T_{P}(\theta ))$, corrected by a term of resistance to chemically induced mortality $\left[1-M_{N}(C(t+T_{P}(\theta ))\right]$. The transtadial passages $\left(D)\to (A_{1}\right)$ and $\left(A_{1})\to (A_{2}\right)$ are modelled in an analogous way with terms of longevity and resistance to chemically induced mortality. *A*_2,*in*_ individuals reach the final stage (*A*_2_) at time $t+T_{E}(\theta )+T_{L}(\theta )+T_{P}(\theta )+T_{D}(\theta )+T_{A_{1}}(\theta )$, and they spend several months reproducing continuously. This suggests structuring the (*A*_2_) by age to characterise their longevity and their contribution, through an age- and temperature-dependent maternity, to the formation of other cohorts, i.e., to the renewal terms $B(t^{\prime }>t)$. If we denote $T_{E-A_{1}}(\theta )=T_{E}(\theta )+T_{L}(\theta )+T_{P}(\theta )+T_{D}(\theta )+T_{A_{1}}(\theta )$ the development time into a reproductive adult (*A*_2_) from birth, then at an age =nΔt counted from $t+T_{E-A_{1}}(\theta )$, the population of *A*_2,*in*_ in the cohort is reduced by a fraction equal to the step Δt, multiplied by the temperature-dependent instantaneous risk $\mu_{A}(\theta ,\text{age})$. At infinity, the population cancels out.

Protonymphs (*P*) and deutonymphs (*D*) are grouped together under the nymph stage (*N*). Similarly, we group the adult stages (*A*_1_) and (*A*_2_) under the adult stage (*A*). These groupings make it possible to unify the modelling of natural and chemical mortality of nymphs (*N*) and adults (*A*) following Equation (5):


$$\begin{array}{c} \left\{ \begin{array}{c} l_{P}\equiv l_{D}\equiv l_{N}\\ M_{P}\equiv M_{D}\equiv M_{N}\\ l_{A_{1}}\equiv l_{A_{2}}\equiv l_{A}\\ M_{A_{1}}\equiv M_{A_{2}}\equiv M_{A} \end{array}\right. \end{array}$$ (5)

The inter-cohort dynamics of *D. gallinae*, schematised in Fig 2, can be described as follows: with the exception of cohorts created during initialisation, a new cohort is generated in the (*E*) stage from the reproductive adults of previous cohorts according to the renewal sub-model. The transtadial passage of individuals is influenced by the probability of induced mortality, which depends directly on the plasma concentration of the treatment in the hens’ blood. In dynamic mode, this concentration is determined by the administration strategy, which in turn is influenced by the total hen population that aggregates all the cohorts present. Lastly, when a cohort sees its individuals reach the reproductive adult stage (*A*_2_), it perceives the current density and, together with other cohorts containing individuals in the (*A*_2_) stage, contributes to reproducing a new cohort according to the renewal sub-model.

**Fig 2:**
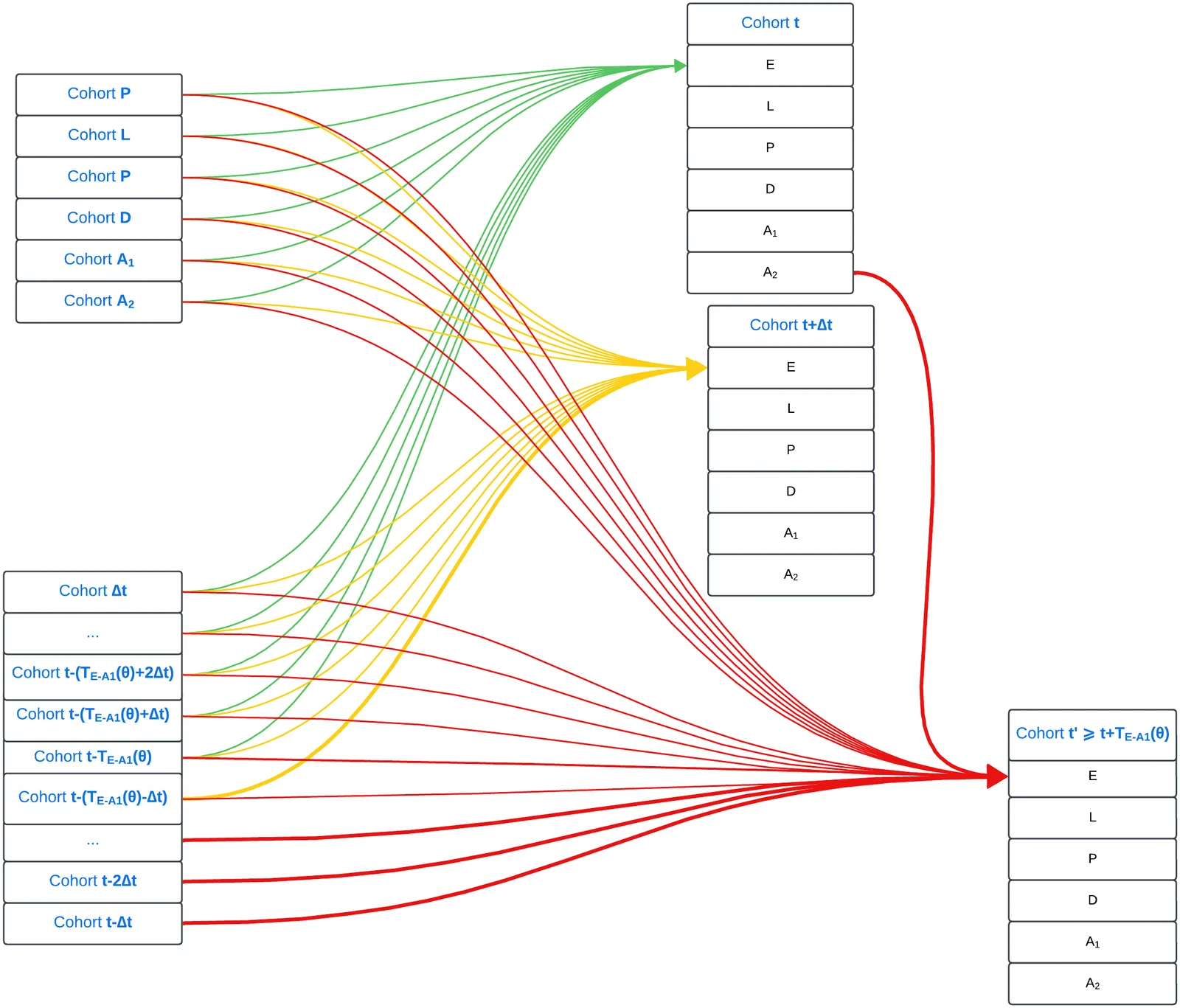
The antecedents and descendants of a cohort born at time *t* > 0. The parents of the individuals in such a cohort come from cohorts some of whose individuals have reached the age $T_{E-A_{1}}(\theta )$ counted from their creation. Similarly, this cohort contributes with its reproductive adults (*A*_2_) to the creation of new cohorts from time $t+T_{E-A_{1}}(\theta )$.

### Sub-models description

Here, we propose a simple sub-model based on interpolation to define, for each temperature θ, the mean durations $T_{A_{1}}(\theta )$, $T_{O}(\theta )$, $T_{E}(\theta )$, $T_{L}(\theta )$, $T_{P}(\theta )$ and $T_{D}(\theta )$.

#### Development time sub-model.

Development time varies with temperature [21]. These durations correspond respectively to the mean times of pre-oviposition (related to the non-reproductive adult stage (*A*_1_)), oviposition, and the egg (*E*), larva (*L*), protonymph (*P*) and deutonymph (*D*) stages (refer to Fig 3).

**Fig 3:**
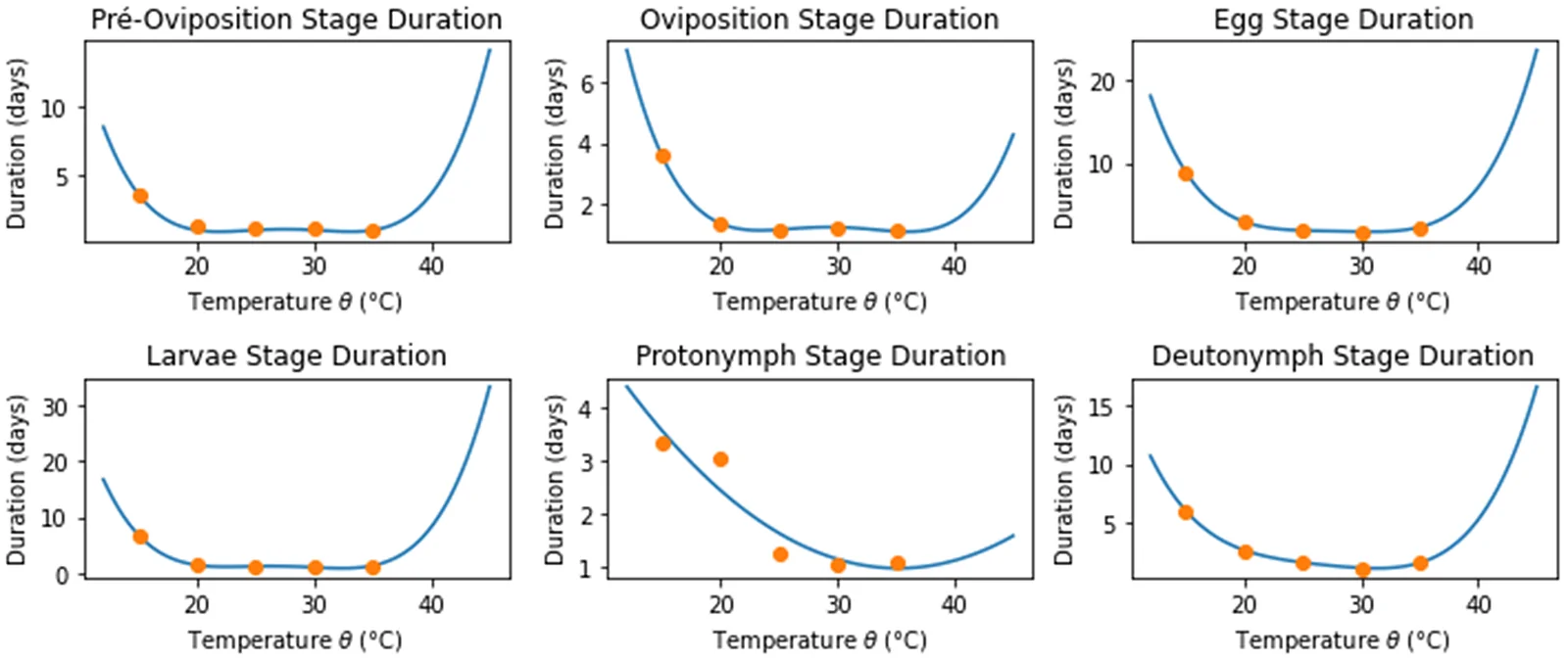
Graphical illustration of the interpolation of average development times as a function of temperature θ.

The fairly convex appearance of the various curves is striking. The apparent symmetry can lead to confusion: the branch on the left reflects the effect of temperature as a kinetic factor, accelerating the development process by shortening it as the temperature rises. On the other hand, the branch on the right is not linked to a kinetic aspect, but rather reflects the degradation of organic matter.

#### Natural mortality sub-model.

For the case of immobile stages (*E*) and (*L*), the difficulty of acquiring the corresponding data, combined with the short development time of these stages (of the order of two days each), compared with the total longevity (which can extend over several months in the absence of treatment), led us to opt for a simple exponential model, with a temperature-dependent parameter θ (Table 3). The associated survival functions are therefore given by Equation (6). We identify the survival probabilities at the right censoring moments $T_{E}(\theta )$ and $T_{L}(\theta )$ with the discrete survival probabilities proposed by [19], which leads to Equation (7). The temperature-dependent instantaneous risks of stages (E) and (L) are thus given by Equation (8). The numerical values of parameters $\alpha_{E}^{\left(1\right)}$, $\alpha_{E}^{\left(2\right)}$, $\alpha_{L}^{\left(1\right)}$, and $\alpha_{L}^{\left(2\right)}$ are given in Table 1.


$$\begin{array}{cc} l_{E}(\theta ,t) & =\exp(-\lambda_{E}(\theta )\;t)\\ l_{L}(\theta ,t) & =\exp(-\lambda_{L}(\theta )\;t) \end{array}$$ (6)

$$\begin{array}{c} \left\{ \begin{array}{cc} l_{E}(\theta ,T_{E}(\theta )) & =\alpha_{E}^{\left(1\right)}-\exp\left(-\alpha_{E}^{\left(2\right)}\;\theta \right)\\ l_{L}(\theta ,T_{L}(\theta )) & =\alpha_{L}^{\left(1\right)}-\exp\left(-\alpha_{L}^{\left(2\right)}\;\theta \right) \end{array}\right. \end{array}$$ (7)

$$\begin{array}{c} \left\{ \begin{array}{cc} \lambda_{E}(\theta ) & =-\frac{1}{T_{E}(\theta )}\;\ln[\alpha_{E}^{\left(1\right)}-\exp\left(-\alpha_{E}^{\left(2\right)}\theta \right)]\\ \lambda_{L}(\theta ) & =-\frac{1}{T_{L}(\theta )}\;\ln[\alpha_{L}^{\left(1\right)}-\exp\left(-\alpha_{L}^{\left(2\right)}\theta \right)]. \end{array}\right. \end{array}$$ (8)

**Table 3:** Numerical values of $\alpha_{E}^{\left(1\right)}$, $\alpha_{E}^{\left(2\right)}$, $\alpha_{L}^{\left(1\right)}$, and $\alpha_{L}^{\left(2\right)}$.

Parameter	Value
$\alpha_{E}^{\left(1\right)}$	0.969
$\alpha_{E}^{\left(2\right)}$	0.269
$\alpha_{L}^{\left(1\right)}$	0.794
$\alpha_{L}^{\left(2\right)}$	0.220

For the case of the movable stages (*N*) and (*A*), thanks to the relative simplicity of handling these stages, experiments have been carried out to study their longevity [10,22]. Fig 4 illustrates the associated results. The curves corresponding to temperatures $\theta =5^{\circ }\text{C}$ and $\theta =45^{\circ }\text{C}$, relative to adults, are merged.

**Fig 4:**
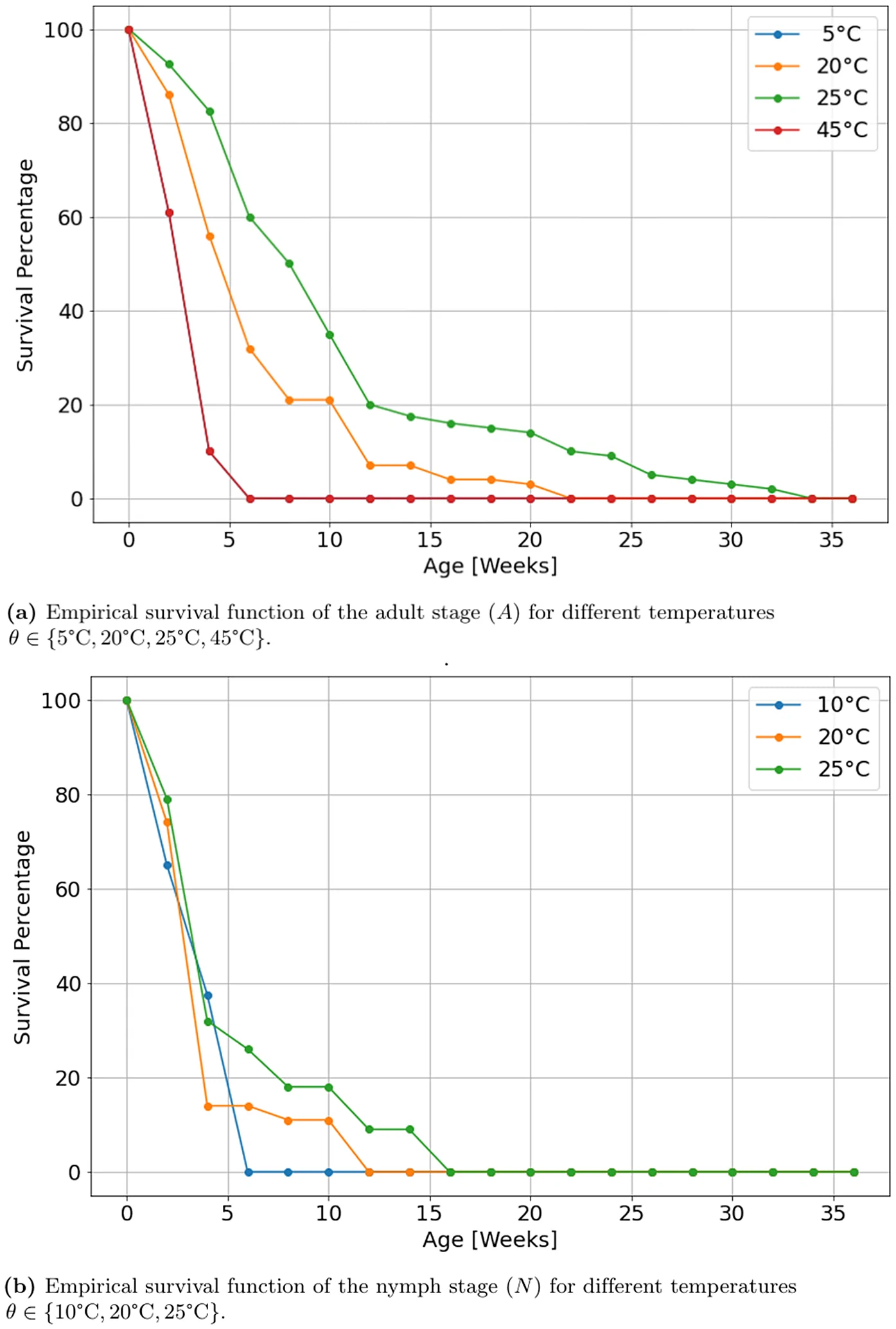
Empirical survival functions for the adult (*A*) and nymph (*N*) stages at different temperatures $\theta \in \{5^{\circ }𝐂,10^{\circ }𝐂,20^{\circ }𝐂,25^{\circ }𝐂,45^{\circ }𝐂\}$.

To assess the significance of ageing effects, we calibrate four models for each (*A*) or (*N*) stage at each available temperature θ: an exponential model, which assumes no sensitivity to ageing; a Weibull model, which incorporates moderate sensitivity to ageing; a Gompertz model, which accounts for intense sensitivity to ageing; and the Gompertz-Weibull model, whose instantaneous risk is constructed by a simple additive weighting of Gompertz and Weibull risks following Equation (9). We choose to compare performance on the basis of the root mean square error RMSE.


$$\begin{array}{c} \mu_{S}(t)=\lambda_{1}\exp(\beta_{1}t)+\lambda_{2}\beta_{2}(\lambda_{2}t{)}^{\beta_{2}-1}. \end{array}$$ (9)

The Table 4 shows that taking moderate account of ageing phenomena via the Weibull distribution can significantly reduce the RMSE. The Gompertz-Weibull presents a particular advantage for temperatures far from the thermal comfort zone of *D. gallinae* (around 25 °C). We decide to continue with the Gompertz-Weibull model. However, at this level, the need to extrapolate the survival model as a function of temperature becomes crucial in order to simplify the results and allow automatic integration into the overall model. We are looking for a function ${\hat{h}}_{S}$ verifying Equation (10) where $\theta_{S}^{*}$ is the optimum temperature for the S∈{N,A} stage. We assume that $\theta_{N}^{*}=\theta_{A}^{*}=\theta^{*}=25^{\circ }\text{C}.$


$$\begin{array}{c} \mu_{S}(\theta ,t)\approx \mu_{S}(\theta_{S}^{*},{\hat{h}}_{S}(t,\theta ,\theta_{S}^{*})),\forall t>0. \end{array}$$ (10)

**Table 4:** RMSE of the Gompertz, Exponential, Weibull, and Gompertz-Weibull models calibrated for the nymphal (*N*) and adult (*A*) stages and different temperatures $\theta {[}^{\circ }C]\in \{5,10,20,25,45\}$.

Stage	θ [°C]	Model	RMSE
Nymph (*N*)	10	Gompertz	5.33 × 10^−2^
		Exponential	5.33 × 10^−2^
		Weibull	2.88 × 10^−2^
		Gompertz-Weibull	3.73 × 10^−2^
	20	Gompertz	5.99 × 10^−2^
		Exponential	5.99 × 10^−2^
		Weibull	4.74 × 10^−2^
		Gompertz-Weibull	4.74 × 10^−2^
	25	Gompertz	5.70 × 10^−2^
		Exponential	5.70 × 10^−2^
		Weibull	3.70 × 10^−2^
		Gompertz-Weibull	3.70 × 10^−2^
Adult (*A*)	5; 45	Gompertz	4.95 × 10^−2^
		Exponential	4.95 × 10^−2^
		Weibull	7.88 × 10^−2^
		Gompertz-Weibull	1.93 × 10^−2^
	20	Gompertz	4.31 × 10^−2^
		Exponential	4.31 × 10^−2^
		Weibull	2.73 × 10^−2^
		Gompertz-Weibull	2.73 × 10^−2^
	25	Gompertz	5.70 × 10^−2^
		Exponential	5.70 × 10^−21^
		Weibull	3.70 × 10^−2^
		Gompertz-Weibull	3.70 × 10^−2^

By definition of the optimum temperature, the further we move away from $\theta^{*}$, whether increasing or decreasing, the greater the instantaneous risk. We assume that this increase is a translation into the future, with a certain time lag proportional to the relative quadratic error of the θ temperature with respect to $\theta^{*}$, which leads to Equation (11). Thus, the temperature-sensitive Gompertz-Weibull model is written via Equation (12). t⩾0 is the time in weeks. $\lambda_{S}^{\left(1\right)}$, $\beta_{S}^{\left(1\right)}$, $\lambda_{S}^{\left(2\right)}$, $\beta_{S}^{\left(2\right)}$ are respectively the parameter values $\lambda_{1}$, $\beta_{1}$, $\lambda_{2}$, $\beta_{2}$ of the Gompertz-Weibull model (9) for the *S* stage at temperature $\theta =\theta^{*}(=25^{\circ }\text{C})$. $\tau_{S}$ is a time constant characteristic of the *S* stage, chosen manually to obtain an acceptable RMSE across the different temperatures. It represents a measure of the stage’s sensitivity to deviation from thermal comfort.


$$\begin{array}{c} {\hat{h}}_{S}(t_{0},\theta ,\theta^{*})=t_{0}+\tau_{S}{\left(\frac{\theta -\theta^{*}}{\theta^{*}}\right)}^{2}. \end{array}$$ (11)

$$\begin{array}{cc} \mu_{S}(t) & =\lambda_{S}^{\left(1\right)}\exp[\beta_{S}^{\left(1\right)}\left(t+\tau_{S}{\left(\frac{\theta -\theta^{*}}{\theta^{*}}\right)}^{2}\right)]\\ & +\lambda_{S}^{\left(2\right)}\;\beta_{S}^{\left(2\right)}[\lambda_{S}^{\left(2\right)}\left(t+\tau_{S}{\left(\frac{\theta -\theta^{*}}{\theta^{*}}\right)}^{2}\right){]}^{\beta_{S}^{\left(2\right)}-1}. \end{array}$$ (12)

Other even powers of $\left(\frac{\theta -\theta^{*}}{\theta^{*}}\right)$ were tested, but the second power proved the most suitable. The numerical values of the parameters of model (12) are summarised in Table 5. The numerical values are expressed in scientific notation in order to represent values of the order of 10^–14^ or less. The RMSE by temperature and by stage are presented in Table 6. As a direct application, we define $T_{S}^{m}(\theta )$ the maximum age of a moving stage S∈{N,A} at temperature θ by Equation (13). We represent, in Fig 5, $T_{A}^{m}(\theta )$ and $T_{N}^{m}(\theta )$ for different values of θ.


$$\begin{array}{c} l_{S}(\theta ,T_{S}^{m}(\theta ))=0,005. \end{array}$$ (13)

**Table 5:** Parameters of the temperature-sensitive Gompertz-Weibull model for the nymph (N) and adult (A) stages.

Parameter	Nymph (N)	Adult (A)
$\lambda_{S}^{\left(1\right)}$	3,00.10^–14^	3,48.10^–15^
$\beta_{S}^{\left(1\right)}$	2,09.10^–1^	9,79.10^–2^
$\lambda_{S}^{\left(2\right)}$	4,70.10^–15^	3,07.10^–16^
$\beta_{S}^{\left(2\right)}$	1,08	1,34
$\tau_{S}$	1800	418

**Table 6:** RMSE of the temperature-sensitive Gompertz-Weibull model for the nymph (N) and adult (A) mobile stages at different temperatures $\theta {[}^{\circ }C]\in \{5,10,20,25,45\}$.

Stage	θ [°C]	RMSE
Nymph (N)	10	0,057003
	20	0,059859
	25	0,043564
Adult (A)	5; 45	0,049374
	20	0,039229
	25	0,037014

**Fig 5:**
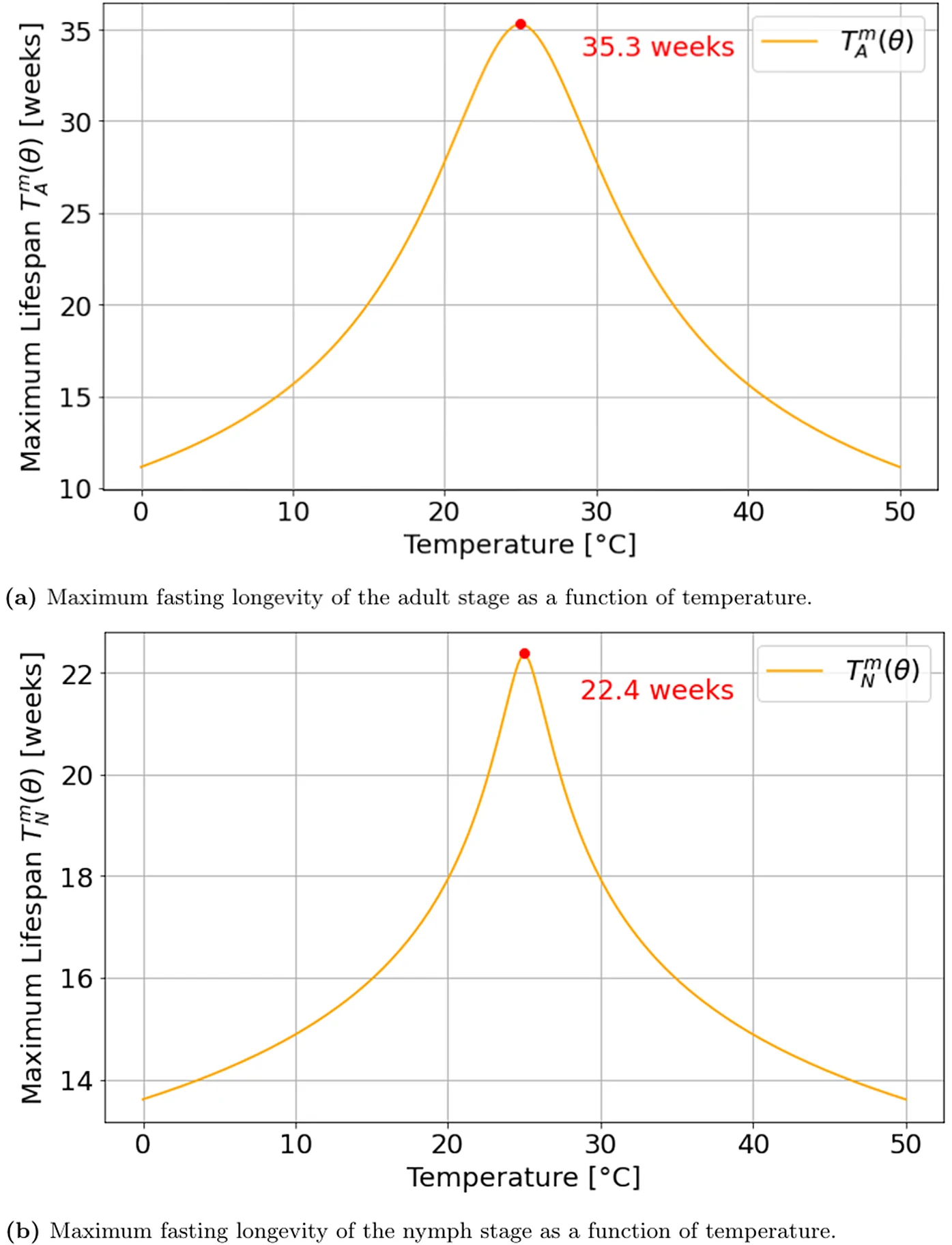
Maximum fasting longevity of the adult (*A*) and nymph (*N*) stages as a function of temperature.

#### Exzolt® pharmacokinetic sub-model.

We begin by defining the plasma concentration of Exzolt® as the sum of the concentrations generated by the different administrations, which gives the Equation (14). Assuming that the doses administered are identical, leads to the simplified Equation (15). $C_{i}^{\left(0\right)}(t)$ represents the temporal evolution of the plasma concentration associated with the dose administered on date $a_{i}$ and is modelled by (16), where $\tau_{1}$ and $\tau_{2}$ are two time constants characterising Exzolt®’s distribution and elimination, respectively. To define our administration strategy, we introduce the dose *varphi* dose by expressing $C_{max}$ in Equation (17) as the product of a default concentration $C_{max}^{0}$, chosen to be the value recommended by MSD, and the dose φ.


$$\begin{array}{c} C(t)=\sum_{i}C^{\left(i\right)}(t-a_{i}). \end{array}$$ (14)

$$\begin{array}{cc} C(t) & =\sum_{i}C^{\left(0\right)}(t-a_{i})\\ & =\sum_{i}C_{i}^{\left(0\right)}(t)\;\text{where}\;C_{i}^{\left(0\right)}(t)=C^{\left(0\right)}(t-a_{i}). \end{array}$$ (15)

$$\begin{array}{c} C_{i}^{\left(0\right)}(t)=\left\{ \begin{array}{cc} 0 & ,\text{ if }t⩽0,\\ C_{\text{max}}\left[1-\exp\left(-\frac{t}{\tau_{1}}\right)\right] & ,\text{ if }0⩽t⩽5\;\tau_{1},\\ C_{\text{max}}\exp\left(-\frac{t-5\tau_{1}}{\tau_{2}}\right) & ,\text{ if }t>5\;\tau_{1}. \end{array}\right. \end{array}$$ (16)

$$\begin{array}{c} C_{max}=\varphi \;C_{max}^{\left(0\right)}. \end{array}$$ (17)

#### Chemical mortality sub-model.

We are interested in modelling the probability of mortality of a nymph (*N*) or an adult (*A*), caused by a transtadial passage to a plasma concentration of Exzolt® *C*. We call these functions $M_{N}(C)$ and $M_{A}(C)$ respectively. Several laboratories have attempted to determine these functions empirically. From the empirical data published by [23], we recognise the form (18) for $M_{S}(C)$ where the stage *S* designate N or A, and the concentration $C_{S}^{*}$ is driven from the respective Lethal Concentration 90% $LC90_{S}$ by the formula (19). The parameters of the pharmacokinetic sub-model and the natural mortality sub-model are summarised in Table 7.


$$\begin{array}{c} M_{S}(C)=1-\exp\left(-\frac{C}{C_{S}^{*}}\right),\;S\in \{N,A\}. \end{array}$$ (18)

$$\begin{array}{c} C_{S}^{*}=\frac{LC90_{S}}{\ln(10)}. \end{array}$$ (19)

**Table 7:** Parameters of the Exzolt® pharmacokinetic and chemical mortality sub-models.

Parameter	Value
$C_{\max}^{\left(0\right)}$	32.77 ppm
$\tau_{1}$	0.3 day
$\tau_{2}$	7.15 days
$C_{A}^{*}$	6.788 ppm
$C_{N}^{*}$	4.960 ppm
φ	Decision variable

For illustrative purposes, we choose to visualise the temporal evolution of the plasma concentration *C*(*t*) (see Fig 6a)) as well as that of the probabilities of induced mortality $M_{A}(C(t))$ and $M_{N}(C(t))$ (see Fig 6b) in response to three administrations of Exzolt®, one week apart, on days *a*_1_ = 28, *a*_2_ = 35, and *a*_3_ = 50. Here, the calendar 𝒜 is the list of instants $a_{1},a_{2}$ and *a*_3_.

**Fig 6:**
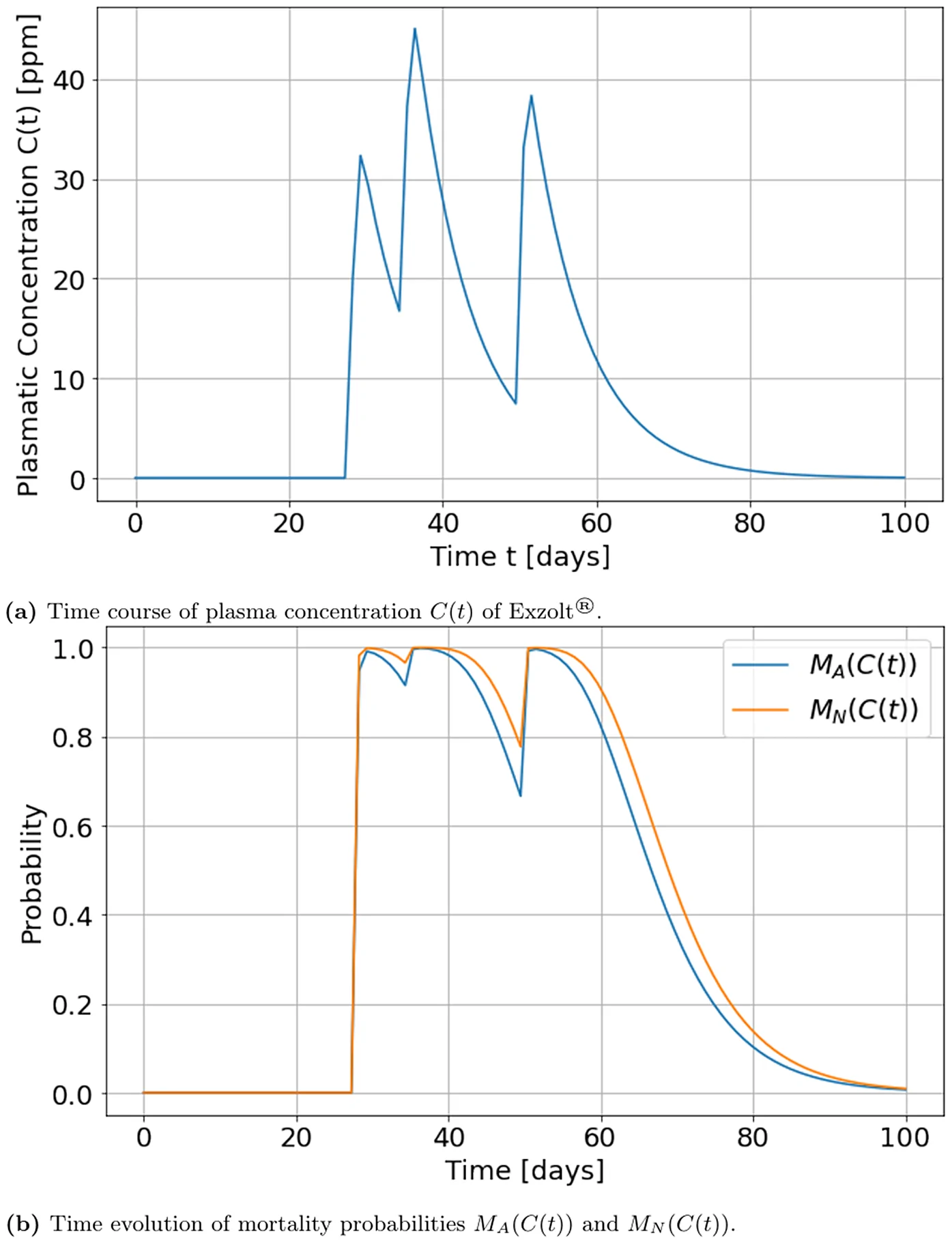
Illustration of the pharmacokinetic sub-model of Exzolt® for three administrations on dates *a*_1_ = 28, *a*_2_ = 35, and *a*_3_ = 50 at the recommended dose.

#### Renewal sub-model.

The renewal of the population takes place through the birth term *B*(*t*) which we decompose according to the Equation (20). $B_{1,\theta }(t)$ is a continuous function that mainly governs the transitory regime of the lice infestation. $B_{2,\theta }(t)$ is a function that mainly governs the steady regime of the lice infestation. K(t)∈[0,1] is a continuous and instantaneous weighting of the two effects $B_{1,\theta }(t)$ and $B_{2,\theta }(t)$, and which is dictated by the current state of the infestation.


B(t)={[(1−K(t)) B1,θ(t)+K(t) B2,θ(t)][1−MA(C(t))],t⩾0,xxxxxxxxxxxxxxxxxxxxxxxxxxxxxxxxx0, otherwise. (20)

$B_{1,\theta }(t)$ is a modified version of Lotka renewal equation given by the Equation (21). The longevity term $l_{E-D}^{Nat}(\theta )$ is given by the Equation (22). For the maternity of *D. gallinae*, we use the empirical law with separate variables proposed by [19]. We chose to retain the numerical values of the parameters $\alpha_{1}$, $\alpha_{2}$, $\alpha_{3}$, $\alpha_{4}$ and $\alpha_{6}$, which proved adequate during the simulations. On the other hand, we decided to cancel the $\alpha_{5}$ parameter, which in reality represents a lag corresponding to the duration of the pre-oviposition, and therefore the non-reproductive adult phase. This phase was not explicitly described by [19], unlike our approach. The numerical values of the maternity function parameters are then given in the Table 8. δ is the sex ratio considered a constant characteristic of the species and equal to 0,5. n(θ) represents the number of cohorts to which the antecedents of the individuals in the cohort born at time *t* > 0 belong and is given by the Equation (25). is the time step, chosen to be one-tenth of a day, as this is the smallest unit that allows distinguishing the durations of the different stages at 20°C. $\tau_{inter}$ is the average interlaying time, approximated to 1 day.


B1,θ(t)={∑k=0n(θ)Δtτinter δ B(t−k Δt) lE−DNat(θ) lA(k Δt) m(θ,k Δt),t⩾0,xxxxxxxxxxxxxxxxxxxxxxxxxxxxxxxxxxx0,otherwise. (21)

$$\begin{array}{c} l_{E-D}^{Nat}(\theta )=l_{E}(\theta ,T_{E}(\theta ))\;l_{L}(\theta ,T_{L}(\theta ))\;l_{P}(\theta ,T_{P}(\theta ))\;l_{D}(\theta ,T_{D}(\theta )). \end{array}$$ (22)

m(θ,age)={m1(θ) m2(age), if age⩾τE−A1(θ),m1(θ) m2(ag0, otherwise. (23)

$$\begin{array}{c} \left\{ \begin{array}{cc} m_{1}(\theta ) & =\alpha_{1}\exp(-\alpha_{2}(\alpha_{3}-\theta {)}^{2}),\\ m_{2}(age) & =\frac{\alpha_{4}(age-\alpha_{5})}{\alpha_{6}^{\left(age-\alpha_{5}\right)}}. \end{array}\right. \end{array}$$ (24)

$$\begin{array}{c} n(\theta )=\frac{T_{A}^{m}(\theta )}{\Delta t}. \end{array}$$ (25)

**Table 8:** Parameters of the *m*-maternity function for *D. gallinae.*

Parameter	Value
$\alpha_{1}$	1.269
$\alpha_{2}$	0.005
$\alpha_{3}$	28.579
$\alpha_{4}$	1.248
$\alpha_{5}$	0
$\alpha_{6}$	1.157

Unlike $B_{1,\theta }(t)$ which is constructed mathematically, the construction of $B_{2,\theta }(t)$ is more computational. A first definition given to $B_{2,\theta }(t)$ by the Equation (26) to guarantee asymptotic stability. The individuals (*E*) that leave the compartment (*E*) at time *t*, whether by transtadial passage or natural mortality, correspond precisely to the individuals born at time $t-T_{E}(\theta )$ and reaching this fa*t*e. This leads to the Equation (27). By applying a unit dose administration, in full permanent regime, we notice that the renewal of the population leads to a new plateau clearly higher than the first one. This is because the information stored by the term $B_{2,\theta }(t)$ is no longer relevant: the population sees its normally maximum value as a simple intermediate value in the growth phase. To remedy this problem, we proceed as follows: We define a parameter called interpolator initialised at None. After calibrating the model, we simulate the dynamics in the absence of treatment over 200 days. We record the set of couples $I_{\theta }$ defined by the Equation (28). By interpolation, we define a function $B_{2,\theta }^{\left(0\right)}(K)$ verifying the Equation (29). We slightly adjust $B_{2,\theta }^{\left(0\right)}(K)$ so that it is strictly increasing. The variable interpolator receives $B_{2,\theta }^{\left(0\right)}(K)$. Eventually, we redefine $B_{2,\theta }(t)$ using the Equation (30).


$$\begin{array}{c} B_{2,\theta }(t)=E_{dead}(t)+E_{out}(t). \end{array}$$ (26)

$$\begin{array}{c} B_{2,\theta }(t)=B(t-T_{E}(\theta )). \end{array}$$ (27)

$$\begin{array}{c} I_{\theta }=\left\{(K(t),B_{2,\theta }(t)):K(t)\in [0,1[\right\}. \end{array}$$ (28)

$$\begin{array}{c} B_{2,\theta }^{\left(0\right)}(K(t))=B_{2,\theta }(t),\;\forall (K(t),B_{2,\theta }(t))\in I_{\theta }. \end{array}$$ (29)

B2,θ(t)={xxB2,θ(0)(K(t)),if K(t)<1 and interpolator≠None,B(t−TE(θ)),if K(t)=1 or interpolator = None. (30)

Individuals of the (*P*), (*D*) and (*A*_1_) stages require a blood meal to make the transtadial passage, while the reproductive adults (*A*_2_) need to renew their energy reserves to maintain their trophic activities. Thus, *K*(*t*) must weight the densities of these stages using the coefficients $\kappa_{P}$, $\kappa_{D}$, $\kappa_{A_{1}}$ and $\kappa_{A_{2}}$. Let *P*_tot_(*t*), *D*_tot_(*t*), *A*_1,tot_(*t*) and *A*_2,tot_(*t*) represent the total populations of the (*P*), (*D*), (*A*_1_) and (*A*_2_) s*t*ages, all cohorts combined. Let $\kappa_{P}$, $\kappa_{D}$, $\kappa_{A_{1}}$ and $\kappa_{A_{2}}$ measure *t*he differences in annoyance levels associated with *t*he different mobile stages. Le*t PDA*(*t*) represent the aggregated resulting measure of the instantaneous annoyance. *PDA*(*t*) is thus given by the Equation (31). We choose to model *K*(*t*) using the Equation (32) where $\kappa_{0}$ is a scale parameter and γ is a shape parameter. On the basis of experiments, we can see that calculating $B_{1,\theta }(t)$ is numerically much more expensive than calculating $B_{2,\theta }(t)$. In addition, *K*(*t*) sometimes stabilises at a value very close to 1 but sligh*t*ly lower, making the more complex calculation superfluous. Consequently, we introduce a Boolean variable, named permanent_forced_regime, which is activated if the following *t*wo conditions are met: K(t−Δt)≠1, indicating tha*t* steady state has not yet been reached, and the last $\left\lfloor \frac{T_{E}(\theta )}{\Delta t}\right\rfloor$ values of *K*(*t*) are equal to three decimal places where ⌊x⌋ denotes the integer part of the real *x*. Once the steady state is forced, *K*(*t*) is systematically rounded to 1, which reduces the execution time of certain simulations by a fac*t*or of 3.


$$\begin{array}{cc} \text{PDA}(t) & =\kappa_{P}\;P_{\text{tot}}(t)+\kappa_{D}\;D_{\text{tot}}(t)\\ & +\kappa_{A_{1}}\;A_{1,\text{tot}}(t)+\kappa_{A_{2}}\;A_{2,\text{tot}}(t). \end{array}$$ (31)

$$\begin{array}{c} K(t)=1-\exp\left(-{\left(\frac{\text{PDA}(t)}{\kappa_{0}}\right)}^{\gamma }\right). \end{array}$$ (32)

#### Infestation control sub-model.

The model presents two usage modes corresponding respectively to the static and dynamic definitions of a control strategy. The choice between the two modes is made using the Boolean variable $\delta_{dyn}$. In static mode ($\delta_{dyn}=0$), the calendar $𝒜=[a_{1},a_{2},a_{3},\ldots ]$ is passed directly as a parameter. This mode is particularly useful for testing traditional control strategies. For example, with 𝒜=[ ], we can observe the dynamics of lice in the absence of treatment. It can also be used to test the strategy recommended by the laboratory, which consists of two administrations with a dose of φ=1, separated by one week.

The dynamic mode ($\delta_{dyn}=1$), on the other hand, constructs the 𝒜 schedule over the course of the simulation. If, at a given moment, the total population of *D. gallinae* exceeds the threshold σ and the last administration took place at least 7 days ago, a new administration is carried out. This new date is then added to 𝒜, and the calculation of the plasma concentration *C*(*t*) in Exzolt® continuously takes into account the updated values of 𝒜.

After each administration, the population of *D. gallinae* systematically begins to decline. Moreover, if the forced steady state is activated, it is deactivated after the administration.

The farmer may only decide to intervene when the infestation is very high, resulting in an initial total population well above our σ threshold. This real situation is not taken into account by the dynamic mode as designed. We therefore decided to define a transitional administration regime, inspired by the experimental protocol proposed by the MSD laboratory, which consists of two administrations one week apart with the recommended dose. Thus, we define our transient regime as follows: as long as the initial total population is less than σ, we administer the φ dose every 7 days.

### Simulations

Before seeking to optimise the control of the infestation, we begin by characterising the dynamics in our building. We set $\delta_{dyn}=0$ to have the static mode and 𝒜=[ ] to have natural dynamics. The ideal simulation time *t* is 630 days. However, as we are going to calibrate the model on a dataset spanning 200 days and the permanent regime is reached around *t*he 110^th^ day, we start with *t* = 200 days with a time step Δt of a tenth of a day. In static mode, *t*he model is insensitive to the numerical values of the parameters φ, $C_{max}^{\left(0\right)}$, σ. We initialise the population in each of the stages: $E_{tot}(t=0)$, $L_{tot}(t=0)$, $P_{tot}(t=0)$, $D_{tot}(t=0)$, *A*_1,*tot*_(*t* = 0), *A*_2,*tot*_(*t* = 0) based on the data from [19]. In their study, 40 laying hens were infested with an initial population of 40,000 *D. gallinae* individuals, distributed as follows: 8 890 adults (A), 8 890 deutonymphs (D), 3 200 protonymphs (P), 4 445 larvae (L), and 14 575 eggs (E). We assume that all the adults in this group are reproductive, i.e., at the (*A*_2_). We normalise the data by the number of laying hens and focus on the dynamics of a single laying hen, assuming all hens are identical. To enable comparison with experimental values from the laboratory, we group the stages (*D*) and (*A*) under the label (*F*), referred to as the’Fed Stages’. As *t*he empirical data correspond to a variable *t*emperature between 10°C and 25°C and a species of *D. gallinae* that is apparently more resistant to cold than the Tunisian one, we decided to calibrate the model manually rather than with an optimisation algorithm so that it would approach the desired behaviour under the guidance of the data rather than sticking to the data. Given that model accepts only constant temperatures, we have chosen θ=20°C which is the indoor temperature in most of the Tunisian poultry buildings. We adjusted the model accordingly. The resulting parameters are given in Table 9. After calibration $B_{2,\theta }^{\left(0\right)}(K(t))$ is determined. The model is therefore prepared to incorporate the treatment effect. First, to validate the model, we simulate the total population over time and compare it to the data of [19]. Second, we simulate the temporal evolution of the proportion of each stage and compare its asymptotic values to that of [19]. Third, we simulate the effect of a single administration at day 120 (𝒜=[120]). Fourth, we switch to dynamic mode ($\sigma_{dyn}=1$). We set $\sigma =\sigma_{min}=$50 000, and simulate control strategies corresponding to $\varphi \in \{0,1;0,25;0,5;1;1,5;1;1,5;2;2,5;3;3,5;4;4,5;5\}\subset [\varphi_{min},$ $\varphi_{max}]$. We introduce the relative algebraic gain in Exzolt®’s purchase cost, denoted ${𝒢}_{CEx}(\varphi ,\sigma )$, defined by Equation (33). ${𝒢}_{CEx}(\varphi ,\sigma )$ measures the algebraic gain (loss if negative) associated with the choice of a dose compared with that recommended at $\left(\sigma ,\varphi )=(\sigma_{min},1\right)$ and, as part of a control strategy starting at the beginning of the breeding cycle. Fifth, we then selected the best value of φ in terms of ${𝒢}_{CEx}(\varphi ,\sigma )$ and simulated control strategies for that dose with different thresholds $\sigma \in [\sigma_{min},\sigma_{max}].$


$$\begin{array}{cc} {𝒢}_{CEx}(\varphi ,\sigma ) & =\frac{CEx(\sigma ,\varphi )-CEx(\sigma_{min},\varphi =1)}{CEx(\sigma_{min},\varphi =1)}\times 100\\ & =\frac{card(𝒜(\sigma ,\varphi ))\;\varphi -card(𝒜(\sigma_{min},1))}{card(𝒜(\sigma_{min},1))}\times 100. \end{array}$$ (33)

**Table 9:** Parameters of the model tested in the absence of treatment.

Group	Parameter	Value
**Initial population**	$E_{tot}(t=0)$	14 575/40
	$L_{tot}(t=0)$	4 445/40
	$P_{tot}(t=0)$	3 200/40
	$D_{tot}(t=0)$	8 890/40
	*A*_1,*tot*_(*t* = 0)	0
	*A*_2,*tot*_(*t* = 0)	8 890/40
**Population dynamics**	$\kappa_{P}$	1
	$\kappa_{D}$	1
	$\kappa_{A_{1}}$	1
	$\kappa_{A_{2}}$	1
	$\kappa_{0}$	8 000
	γ	0.6
	δ	0.5
	$\sigma_{\max}$	225 000
	interpolator	None
**Environment**	*t*	200 days, 0.1-day intervals
	θ	$20{\;}^{\circ }C$
**Dynamic mode**	$\delta_{dyn}$	0
	𝒜	[]
	φ	1
	$C_{\max}^{\left(0\right)}$	32.77 ppm
	σ	$5\times 10^{4}$

## Results

### Population dynamics

In Fig 7, we isolate the simulated total population in blue and the observed total population in red. During the first two months (60 days), when temperatures are around 20°C, the model faithfully reproduces the empirical data. However, after this period, the actual temperature drops significantly, reaching around 10°C due to a cold snap. Naturally, we expect our model, which does not take account of this drop in temperature, to overestimate the data. Furthermore, the model is calibrated to reach a steady state around day 110. Although it reaches this regime more quickly than the empirical data, the two curves eventually converge asymptotically from day 150. Thus, at first sight, the model seems promising for modelling population dynamics in dark buildings. We propose to deepen the validation by exploring the stage composition of the total population simulated under a hypothetical constant temperature of $\theta =20^{\circ }\text{C}$. Fig 8 the temporal evolution of this composition and compare, in Table 10, the asymptotic state obtained with that observed under a variable temperature $\theta \in [10^{\circ }\text{C},25^{\circ }\text{C}]$. For stage $\left(S)\in \{(E),(L),(P),(D),(A_{1}),(A_{2})\right\}$, let $r_{\theta }^{\left(S\right)}(+\infty )$ denote its proportion in the asymptotic composition of the total population. $r_{\theta }^{\left(F\right)}(+\infty )$ is therefore given by the sum of $r_{\theta }^{\left(S\right)}(+\infty )$, $r_{\theta }^{\left(S\right)}(+\infty )$, and $r_{\theta }^{\left(S\right)}(+\infty )$. We deduce that, although the model does not explicitly take into account the variation in temperature observed in the empirical data, it tends to approximate the asymptotic composition of the simulated population satisfactorily.

**Table 10:** Asymptotic composition of the total population simulated under a hypothetical constant temperature $\theta =20^{\circ }𝐂$ vs that observed under a variable temperature $\theta \in [10^{\circ }𝐂,25^{\circ }𝐂]$.

Stage	Asymptotic proportion	% simulated	% real
(*E*)	$r_{\theta }^{\left(E\right)}(+\infty )$	23,57	30,02
(*L*)	$r_{\theta }^{\left(L\right)}(+\infty )$	11,72	11,66
(*P*)	$r_{\theta }^{\left(P\right)}(+\infty )$	14,73	13,88
(*F*)	$r_{\theta }^{\left(F\right)}(+\infty )$	49,98	44,44

**Fig 7:**
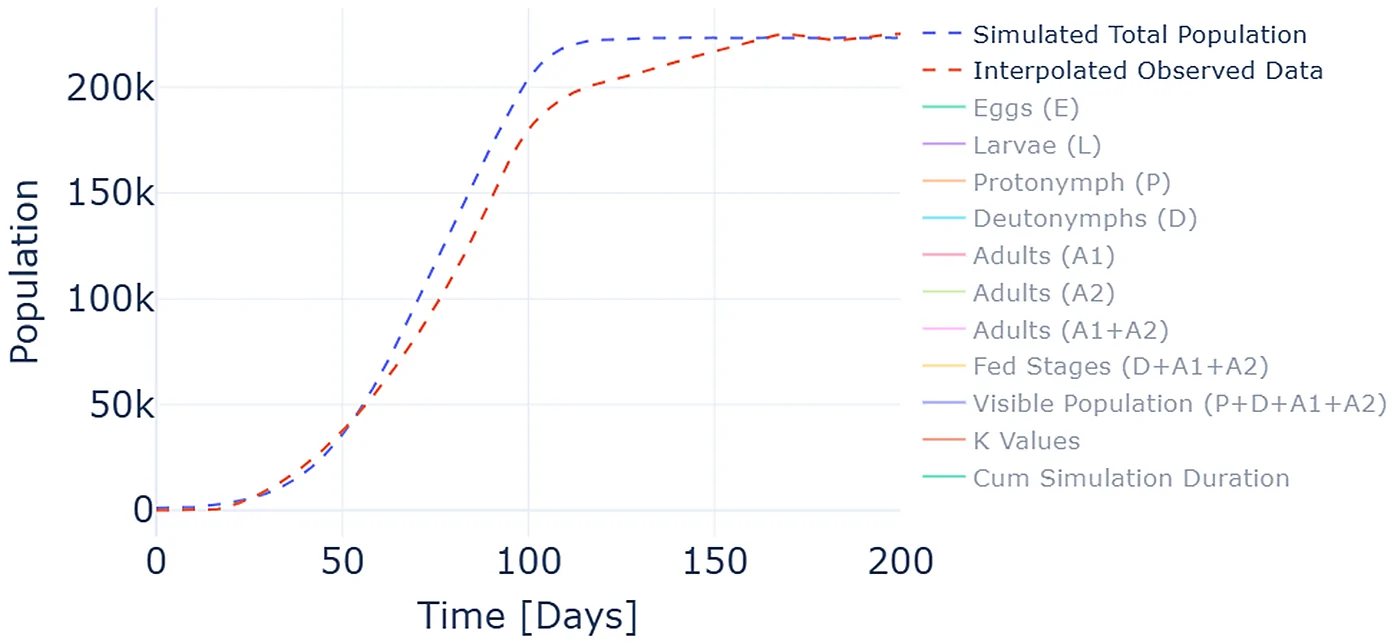
Total population simulated under a hypothetical constant temperature $\theta =20^{\circ }𝐂$ versus total population observed under a variable temperature $\theta \in [10^{\circ }𝐂,25^{\circ }𝐂]$.

**Fig 8:**
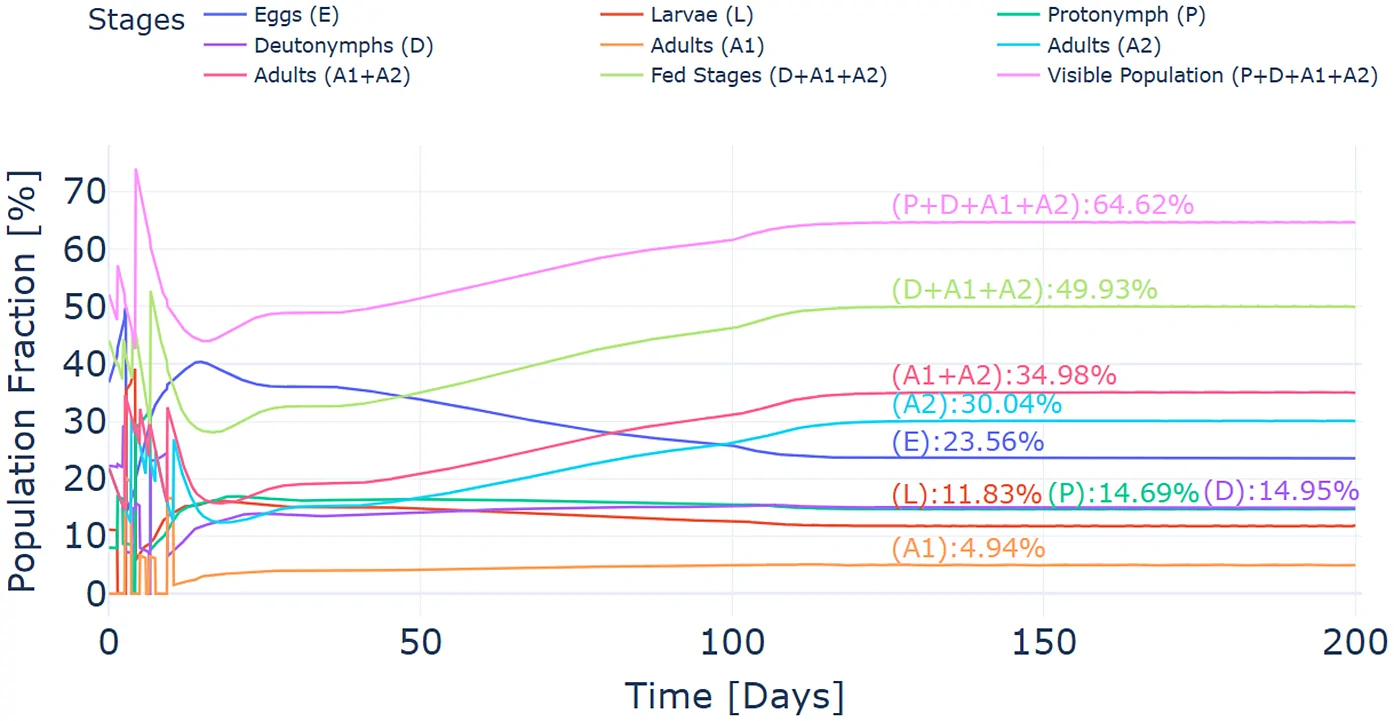
Temporal evolution of the stadial composition of the total population simulated with the model in the absence of treatment and at a temperature of $\theta =20^{\circ }𝐂$.

### Effect of a single administration at the recommended dose

In order to validate the pharmacokinetic and chemical mortality sub-models, we proceed with the second simulation keeping exactly the same set of parameters as in the Table 9, with the exception of the administration schedule being changed to 𝒜=[120]. Following this administration, and as shown in Fig 9, the total population decreased by 87.03% and took approximately 10 weeks to return to its maximum value. The results are similar to those obtained in the simulation by Huber et al (2010), confirming the validity of our approach.

**Fig 9:**
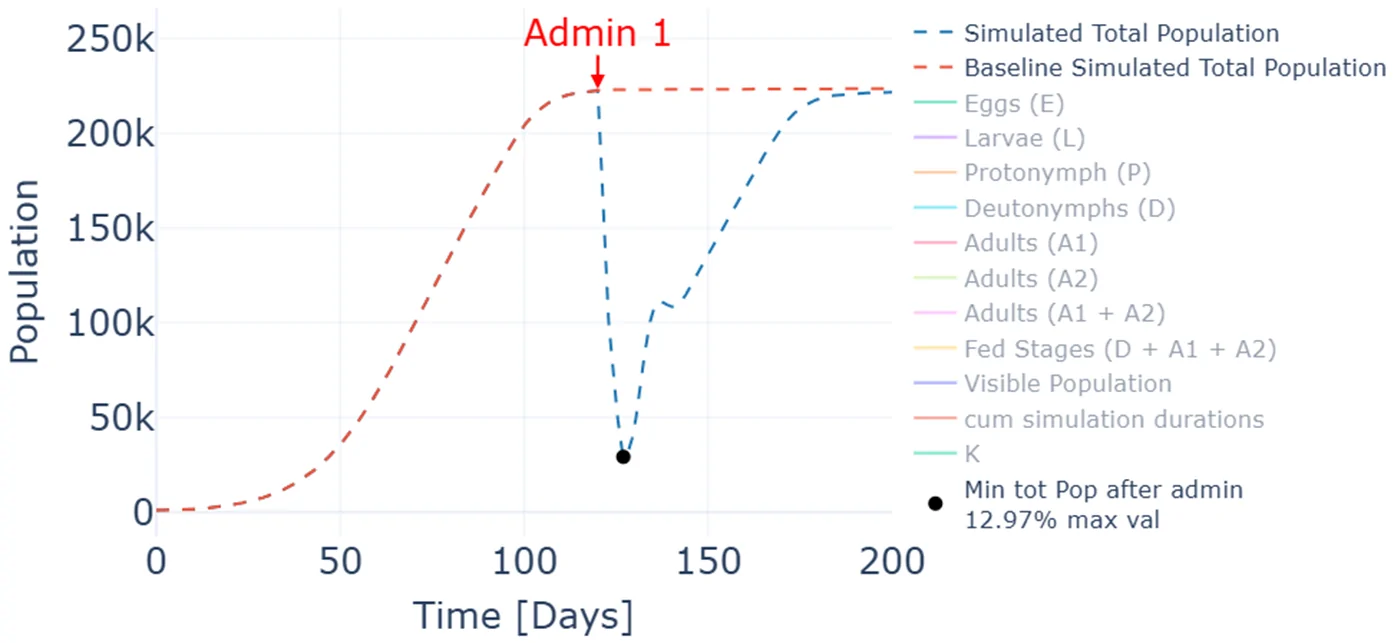
Temporal evolution of the dynamics of *D. gallinae* simulated at a constant temperature $\theta =20^{\circ }𝐂$ in response to an administration of Exzolt® on day 120 with the recommended dose φ=1.

### Dose selection

In Table 11 we present the lists $𝒜(\sigma =\sigma_{min},\varphi )$ as a function of the dose φ. In order to aggregate the information in Table 11, we decided to evaluate the evolution of the series of durations $(\Delta a_{i}{)}_{i}$ between two successive administrations, applied respectively to $a_{i}$ and $a_{i+1}\in 𝒜$. To these durations, we apply the integer part operator ⌊·⌋ to facilitate management by farmers. This approach takes into account the residual effects of previous administrations in determining future administrations. We present the results in Fig 10. We note that from the 3^rd^ administration onwards, the cumulative effect stabilises and the $(\Delta a_{i}(\sigma ,\varphi ){)}_{i}$ series becomes stationary. We then define the asymptotic inter-administration interval, denoted $\Delta a^{\left(+\infty \right)}(\sigma ,\varphi )$, which naturally depends on the φ dose and the σ threshold. $\Delta a^{\left(+\infty \right)}(\sigma ,\varphi )$ can serve as a simplified frequency guideline provided to farmers, making it easier for them to manage administration schedules.

**Table 11:** Administration schedule A(σ,φ) to maintain an infestation of less than $\sigma =\sigma_{min}=50\;000$ individuals of *D. gallinae* per laying hen for different doses φ expressed according to that recommended by the manufacturer laboratory. Dates are counted from the start of the breeding cycle. The initial infestation is below the σ threshold.

φ	$𝒜(\sigma =\sigma_{min},\varphi )$
0,1	[56; 67,8; 78,2; 87,8; 97,2; 106,6; 116,1; 125,7; 135,4; 145,2; 155; 164,8; 174,6; 184,4; 194,2; 204; 213,8; 223,6; 233,4; 243,2; 253; 262,8; 272,6; 282,4; 292,2; 302; 311,8; 321,6; 331,4; 341,2; 351; 360,8; 370,6; 380,4; 390,2; 400,00; 409,80; 419,60; 429,40; 439,20; 449,00; 458,80; 468,60; 478,40; 488,20; 498,00; 507,80; 517,60; 527,40; 537,20; 547,00; 556,80; 566,60; 576,40; 586,20; 596,00; 605,80; 615,60; 625,40]
0,25	[56; 75,5; 93,9; 112,8; 132,1; 151,5; 170,9; 190,3; 209,6; 229; 248,3; 267,7; 287,1; 306,4; 325,8; 345,1; 364,5; 383,9; 403,2; 422,6; 441,9; 461,3; 480,7; 500; 519,4; 538,7; 558,1; 577,5; 596,8; 616,2]
0,5	[56; 81,1; 106,2; 131,8; 157,5; 183,2; 208,9; 234,6; 260,3; 286; 311,7; 337,4; 363,1; 388,8; 414,5; 440,2; 465,9; 491,6; 517,3; 543; 568,7; 594,4; 620,1]
1	[56; 87,00; 118,70; 150,80; 182,90; 215,00; 247,10; 279,20; 311,30; 343,40; 375,50; 407,60; 439,60; 471,70; 503,80; 535,90; 568,00; 600,10]
1,5	[56; 90,4; 125,9; 161,7; 197,4; 233,1; 268,9; 304,6; 340,3; 376; 411,7; 447,5; 483,2; 518,9; 554,6; 590,3; 626,1]
2	[56; 92,9; 131,1; 169,5; 207,8; 246,1; 284,4; 322,7; 361; 399,3; 437,6; 475,9; 514,2; 552,5; 590,8; 629,1]
2,5	[56; 94,9; 135,2; 175,5; 215,8; 256,1; 296,4; 336,7; 377; 417,3; 457,6; 497,9; 538,2; 578,5; 618,8]
3	[56; 96,5; 138,5; 180,4; 222,4; 264,3; 306,2; 348,1; 390,1; 432; 473,9; 515,9; 557,8; 599,7]
3,5	[56; 97,9; 141,3; 184,6; 227,9; 271,2; 314,5; 357,8; 401,1; 444,4; 487,7; 531,1; 574,4; 617,7]
4	[56; 99,1; 143,7; 188,2; 232,7; 277,2; 321,8; 366,3; 410,8; 455,3; 499,8; 544,3; 588,8]
4,5	[56; 100,1; 145,9; 191,4; 237; 282,6; 328,2; 373,7; 419,3; 464,9; 510,5; 556; 601,6]
5	[56; 101,1; 147,8; 194,3; 240,8; 287,4; 333,9; 380,4; 426,9; 473,5; 520; 566,5; 613]

**Fig 10:**
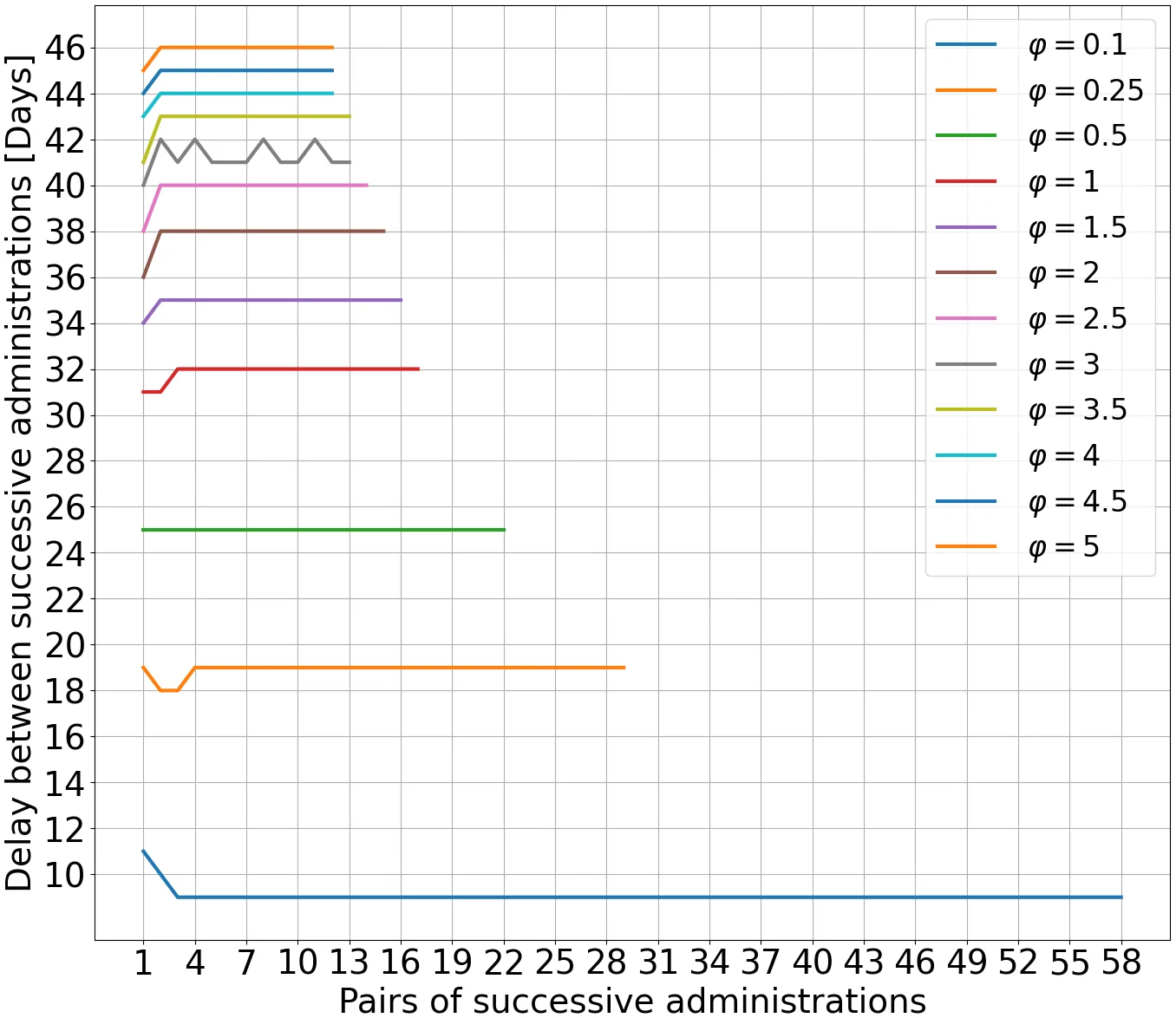
Evolution of the time lag between two successive administrations of a schedule $𝒜(\sigma =\sigma_{min}=50000,\varphi )$ for different φ doses. The *i*^th^ pair designates the *i*^th^ and $(i+1{)}^{th}$ administrations. Dates are counted from the start of the breeding cycle. The initial infestation is below the σ threshold.

Table 12 shows that a reduction in dose is generally associated with an increased number of administrations. This approach is more cost-effective, as shown by the positive algebraic gain obtained with lower doses. This optimises treatment costs while respecting the biological needs of the hens. Fig 11 graphically illustrates these results, providing a clear visualisation of the relationships between dose, inter-administration interval and algebraic gain. Here, we have decided to continue with the dose φ=0.1 and to modulate the threshold. The frequency of administration will necessarily be greater than $\Delta a^{\left(+\infty \right)}(\sigma_{min};\varphi =0,1)=9$ days, a threshold which we already consider relevant in a practical sense.

**Table 12:** Comparison of strategies defined by the threshold $\sigma =\sigma_{min}$ and the dose φ for the control of infestation at the beginning of the breeding cycle, as a function of the asymptotic inter-administration interval $\Delta a^{\left(+\infty \right)}$ [days], the number of administrations card(𝒜), and the algebraic gain in Exzolt® purchase cost ${𝒢}_{CEx}$ compared to the reference strategy with φ=1. See Fig 11 for a graphical representation of the results.

φ	$\Delta {𝐚}^{\left(+\infty \right)}$[days]	card(𝒜)	${𝒢}_{CEx}$
0,1	9	59	+67,22%
0,25	19	30	+58,33%
0,5	25	23	+36,11%
1	32	18	0,00%
1,5	35	17	−41,67%
2	38	16	−77,78%
2,5	40	15	−108,33
3	42	14	−133,33%
3,5	43	14	−172,22%
4	44	13	−188,89%
4,5	45	13	−225,00%
5	46	13	−261,11%

**Fig 11:**
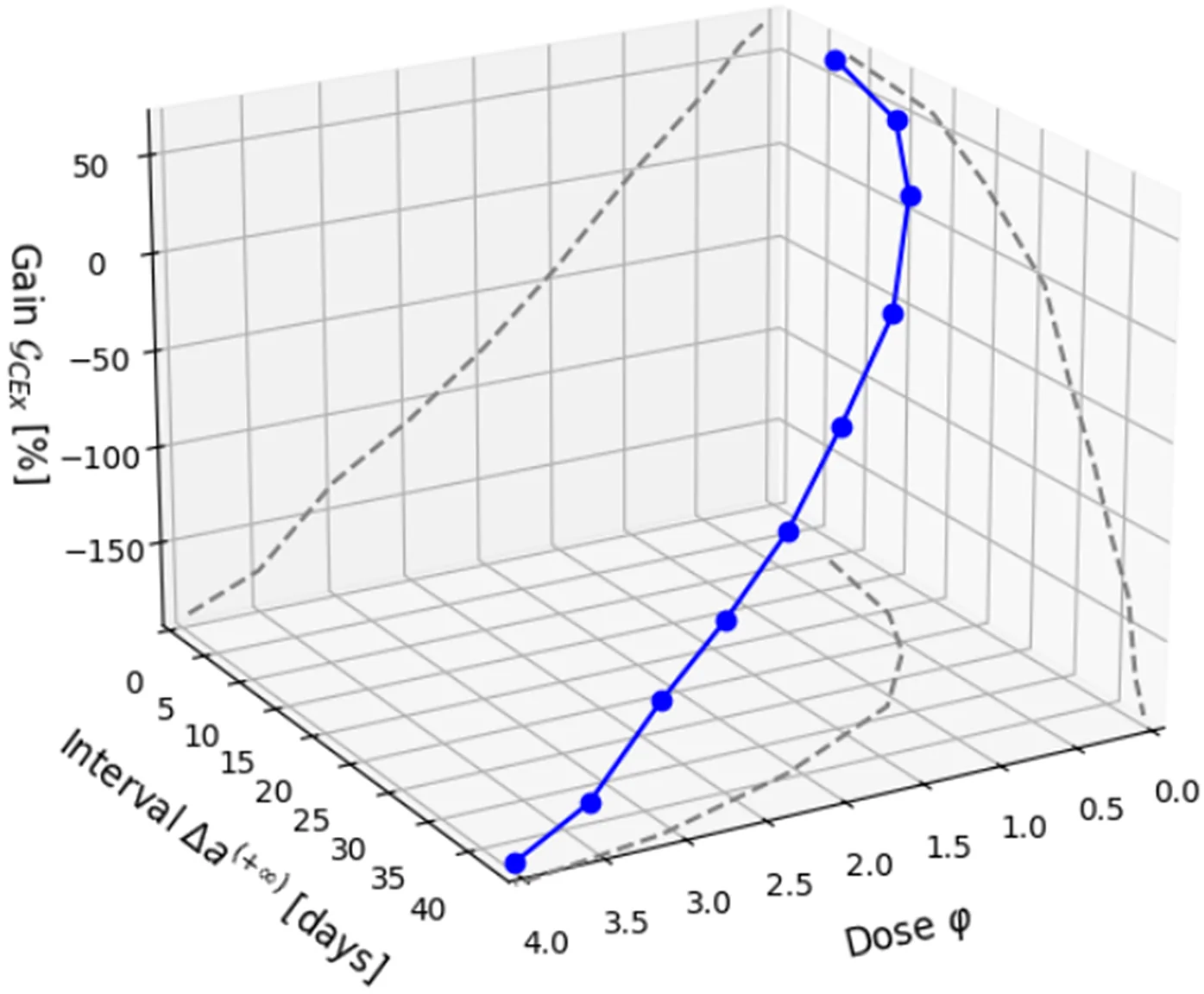
Variation of the asymptotic inter-administration interval $\Delta a^{\left(+\infty \right)}$ [days] and of the algebraic gain ${𝒢}_{CEx}$ for a strategy defined by $\sigma =\sigma_{min}=50\;000$ as a function of the dose φ. 3D representation with projections onto the three corresponding planes. See Table 12 for values.

### Threshold selection

After setting the dose to one tenth of the recommended dose, the problem (4) reduces to (34). To refine the mathematical definition of the problem and propose an analytical solution, we aim to characterise CEx(σ,φ=0.1). To achieve this, we simulate various scenarios by varying the values of σ while keeping the dose fixed at φ=0,1. We then calculate the corresponding number of administrations, card(𝒜(σ,φ=0.1)). The resulting curve is presented in Fig 12. The affine elongation of the points suggests approximating card(𝒜(σ,φ=0,1)) by an affine line dependent on the threshold and the dose, which cancels out at $\sigma =\sigma_{max}$ regardless of the dose. card(𝒜(σ,φ=0,1)) is thus given by Equation (35). $v(\sigma_{max},\varphi )$ is calculated by evaluating card(𝒜(σ,φ)) for $\sigma =\sigma_{max}$. In particular, $v(\sigma_{max};\varphi =0,1)$ is given by Equation (36). CEx(σ,φ) and 𝒞(σ,φ) are consequently given by equations (37) and (38). The resulting optimal threshold, solution $\sigma^{*}(\varphi )$ of the problem (34), is given by Equation (39). In particular, $\sigma^{*}(\varphi =0,1)\approx 146430$. Fig 13 summarises the resolution of the threshold selection problem.


$$\begin{array}{c} \underset{\sigma_{min}⩽\sigma ⩽\sigma_{max}}{\min}𝒞(\sigma ;\varphi =0,1). \end{array}$$ (34)

$$\begin{array}{c} card(𝒜(\sigma ,\varphi ))=-\frac{v(\sigma_{max},\varphi )}{\left(\sigma_{max}-\sigma_{min}\right)}(\sigma -\sigma_{min})+v(\sigma_{max},\varphi ). \end{array}$$ (35)

$$\begin{array}{c} v(\sigma_{max};\varphi =0,1)=card(𝒜(\sigma =\sigma_{min},\varphi =0,1))=56,48. \end{array}$$ (36)

$$\begin{array}{cc} CEx(\sigma ,\varphi ) & =\left[\frac{-v(\sigma_{max},\varphi )}{\left(\sigma_{max}-\sigma_{min}\right)}(\sigma -\sigma_{min})+v(\sigma_{max},\varphi )\right]\\ & \times \alpha_{CEx}\;\varphi . \end{array}$$ (37)

$$\begin{array}{cc} 𝒞(\sigma ,\varphi ) & =CC+\frac{CDg(\sigma_{max})}{(\sigma_{max}-\sigma_{min}{)}^{2}}(\sigma -\sigma_{min}{)}^{2}\\ & +\alpha_{CEx}\varphi \left[-\frac{v(\sigma_{max},\varphi )}{\left(\sigma_{max}-\sigma_{min}\right)}(\sigma -\sigma_{min})+v(\sigma_{max},\varphi )\right]. \end{array}$$ (38)

$$\begin{array}{cc} \sigma^{*}(\varphi ) & =\sigma_{min}+\frac{\alpha_{CEx}\;\varphi \;v(\sigma_{max},\varphi )}{2\;CDg(\sigma_{max})}(\sigma_{max}-\sigma_{min}). \end{array}$$ (39)

**Fig 12:**
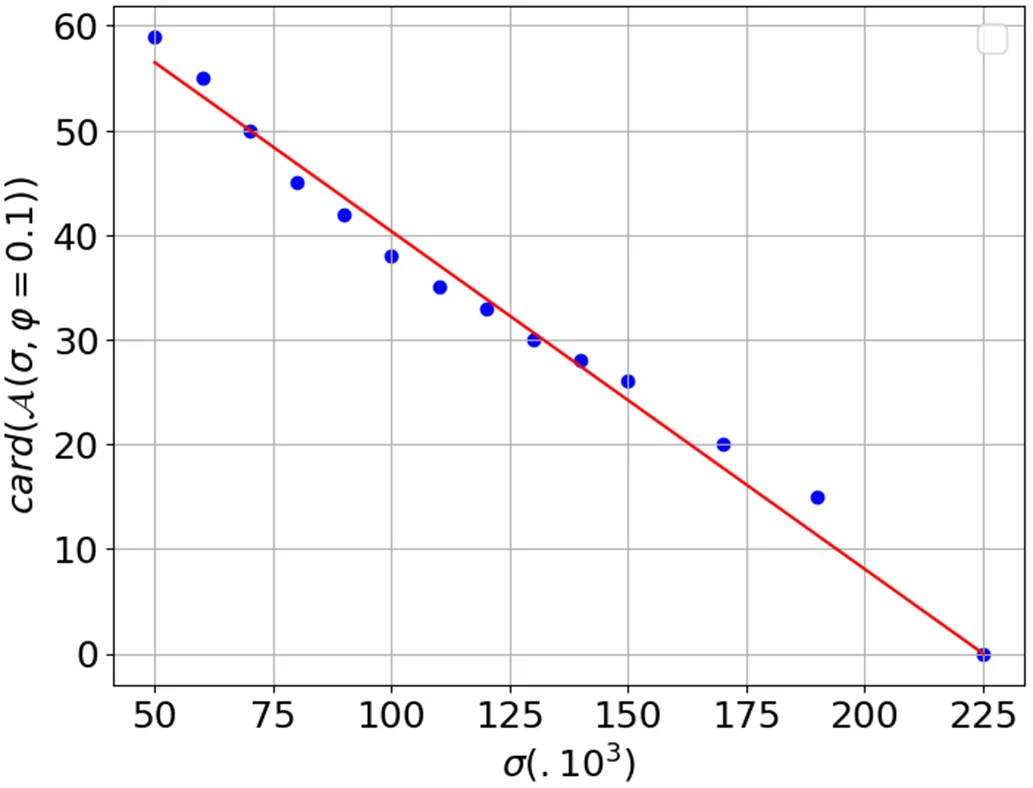
Evolution of the number of administrations card(𝒜(σ,φ=0,1)) as a function of σ for a dose φ=0,1. The dose is expressed relative to that recommended by the manufacturer.

**Fig 13:**
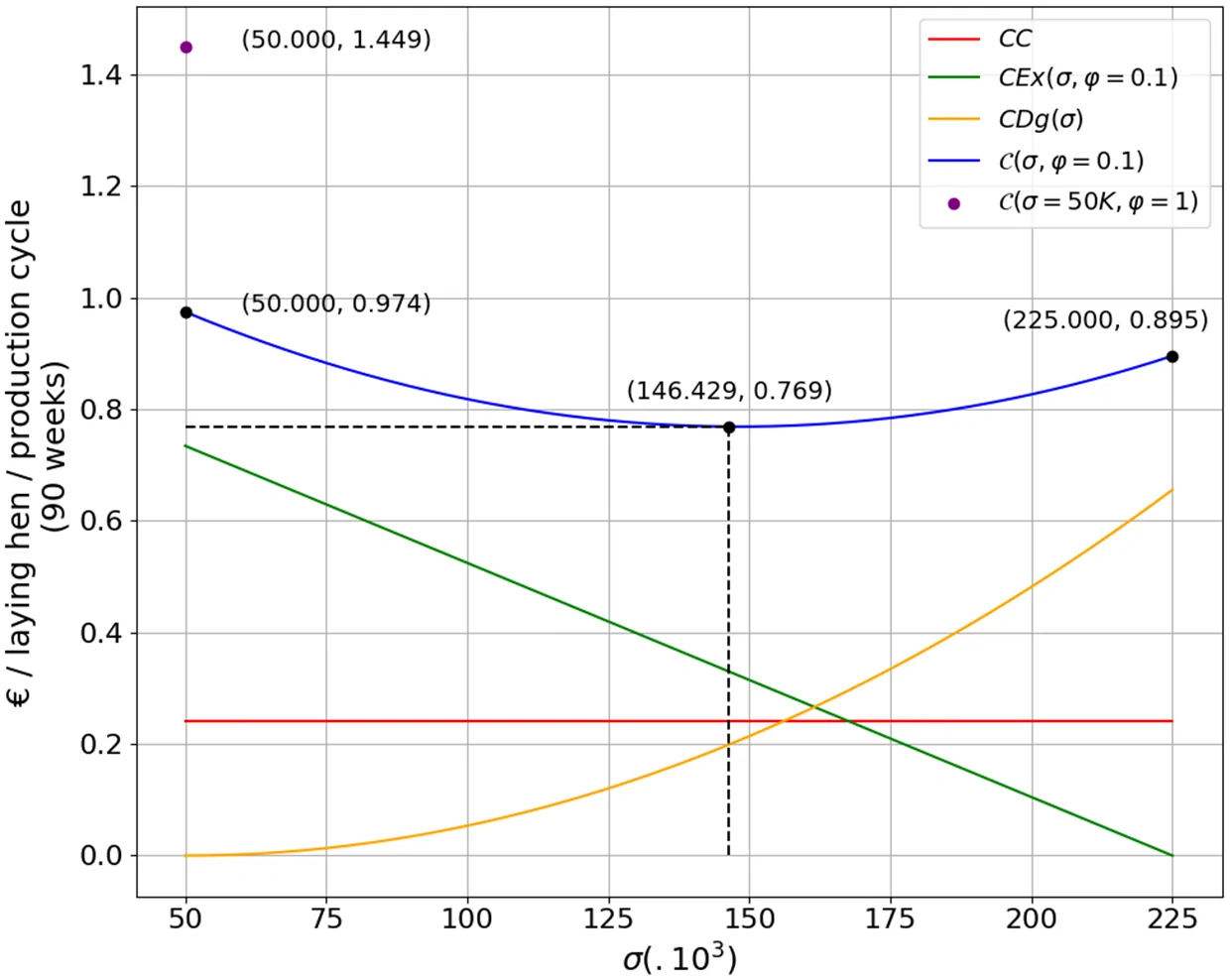
Variation of the different costs per laying hen over a 90-week production cycle, associated with a strategy defined by a dose φ=0,1 and a tolerance threshold σ of the total population as a function of σ. The point at the far right represents the traditional control strategy, where Exzolt® is not applied.

We observe that the moderate or even low level of infestation significantly reduces the potential profit margin of the treatment. Indeed, the traditional strategy, which relies solely on alternative control measures without applying Exzolt® ($\sigma =\sigma_{max}=225\;000$), appears to be a quite plausible option. The strategy defined by the threshold $\sigma =\sigma_{min}$ and the recommended dose φ=1 incurs a cost 61.90% higher than the reference strategy without treatment. Even when reducing the dose to φ=0.1, losses are only reduced to 8.83%, which remains insufficient. The shape of the blue curve representing the total cost suggests an increasing interest in applying the treatment as the infestation level rises.

With an optimal choice of σ, we save €0.126 per laying hen over a 90-week production cycle, corresponding to a 14.15% reduction in total cost and a 19.53% reduction when excluding the constant component *CC*, compared to a traditional system (reference strategy without treatment). The required number of administrations can be estimated using Equation (35), yielding $card(𝒜(\sigma^{*}(\varphi =0,1),\varphi =0,1))\approx 39$ administrations. To estimate the schedule, the simplest method is to divide the total cycle duration into 39 intervals of approximately $\frac{630}{39}\approx 16$ days each. For greater accuracy, the simulation can be rerun with the parameter $\sigma^{*}$.

## Discussion

In this study, we used a single treatment with Exzolt® (Fluralaner), the only acaricide currently authorized for the control of *D. gallinae* in laying hens, which does not require a withdrawal period for eggs intended for human consumption. The aim of our work is not to assess the efficacy of this drug, but to use it as a case study to explore the functionalities of our model, which could be applied to optimise control strategies against *D. gallinae* and to identify, in silico, new potential protocols which aim to preserve long-term acaricide efficacy and minimise ecological disruption. For this reason, we intentionally selected a single Exzolt ® treatment as the reference protocol.

### Model performance & calibration

The model was calibrated using the experimental data of [19], representing an average infestation scenario under controlled conditions. It accurately replicated population growth during the first 60 days under stable temperatures (≈20°C). While a deviation occurred after a sharp temperature drop to 10°C – a variable not dynamically incorporated- the model’s predictions converged asymptotically with observed data by day 150. This convergence confirms the robustness of the core biological parametrization and supports the model’s utility for long-term simulations in dark poultry houses [7,19].

### Temperature-dependent development and mortality

A significant advancement of our model is the explicit incorporation of ambient temperature as a critical environmental driver of mite development and mortality. The development times derived from our interpolation sub-model are consistent with published experimental data: [21] demonstrated that life cycle duration decreases from 28 days at 15°C to 6 days at 30°C, with optimal development at 30°C and adverse effects emerging at 35°C. [10] further established that oviposition, hatching and survival are maximized between 25 and 30°C at 70% relative humidity. Our Gompertz-Weibull sub-model for mobile stages (nymphs and adults) proved particularly advantageous for temperatures outside the thermal comfort zone (25°C). The second-power transformation of temperature deviation (τ) effectively captured the increase in instantaneous mortality risk as temperatures diverge from the optimum, significantly reducing root mean square error (RMSE) compared to simpler exponential or Weibull models. This formulation acknowledges that temperature acts not only as a kinetic accelerator within the optimal range but also as a stressor at extremes, reflecting the degradation of organic matter and physiological function at high temperatures [24].

### Comparaison with existing models

Our model advances existing frameworks for D. gallinae population dynamics in several critical ways. The foundational stage-structured model of [19] accurately described temperature-dependent development, and the theoretical model of [18] evaluated control efficacy through simulations. [25] characterised the genetic structure and dispersal patterns of PRM, confirming the strictly anthropogenic origin of between-farm circulation and the high persistence of mites even during sanitary breaks. Furthermore, the work of [26] on advancing IPM for *D. gallinae* emphasized the necessity of “scientifically sound warning, forecasting and early diagnosis systems” and identified six requirements for operational population models. Our model satisfies these requirements by: (1) requiring minimal staff input (temperature and monitoring data), (2) providing easily interpretable outputs (treatment schedules and cost projections), (3) compensating for location- and time-specific variables through recalibration, (4) enabling identification of infestation hotspots through cohort structuring (5) forecasting treatment efficacy, and (6) accommodating different monitoring methods. In practice, our model has the potential to predict the population expansion dynamics of *D. gallinae* on a given farm and determine when densities exceed economically harmful thresholds, thereby justifying acaricidal treatments.

### Pharmacokinetics coupling and treatment simulation

In our model, we coupled for the first time, detailed mite population dynamics with the pharmacokinetics of Fluralaner (Exzolt®) and a straightforward economic optimisation framework that incorporates drug costs. The choice of Fluralaner Exzolt®) for this analysis was motivated by two key characteristics: it is currently the only acaricidal product approved for use in laying hens that does not leave residues in eggs nor adversely affect bird health and productivity [27], and the extensive availability of pharmacokinetic and efficacy data enabled robust parametrization [28]. Nevertheless, the model’s structure is inherently adaptable; its framework can be recalibrated to simulate the efficacy and economics of other acaricides, provided sufficient pharmacological and toxicity data are available, making it a versatile tool for evaluating future treatment options [6]. The integration of a Fluralaner pharmacokinetic sub-model represents a novel contribution to PRM modelling. Our simulated single administration at the recommended dose resulted in an 87.03% reduction in total mite population, with full rebound occurring after approximately 10 weeks. This finding aligns with field efficacy data reported for Fluralaner [18,29] and validates the coupled pharmacokinetic and chemical mortality sub-models. The initial in silico results from our model support a potential paradigm shift from eradication-focused strategies to sustainable, threshold-based population management. This approach aligns with core IPM principles, which aim to preserve long-term acaricide efficacy and minimise ecological disruption [30,31].

### Economic optimisation and threshold-based management

Our primary in silico finding are suggesting a strategy combining substantially reduced Fluralaner doses (φ=0.1 of the recommended dose) with high-frequency applications, guided by a farm-specific infestation threshold (σ). The model quantified an optimal economic threshold of σ≈164 430 mites per hen. To maintain a population below this level, the model prescribes approximately 39 administrations over a 90-week cycle, equating to a treatment roughly every 16 days, with intervals stabilizing after the initial three administrations (e.g., at 9 days for φ=0.1 and $\sigma =\sigma_{min}$). Our model indicates that this strategy is economically superior: while reducing the dose increases the number of administrations, it significantly improves cost efficiency, achieving up to a 14.15% reduction in total cost (19.53% excluding fixed costs) compared to a no-acaricide baseline, and proving more cost-effective than using the full recommended dose protocol ([2,32]). The derived algebraic gain curves have the potential to serve as a valuable in silico decision-support tool, enabling the identification of potential candidate protocols that balance cost, labour and efficacy for further field validation. However, the number of treatments (39) and the optimal threshold (146 430 mites per laying hen) should be regarded as experimental, putative outputs derived from the model calibrated with a specific dataset [19]. Preliminary, these values demonstrate the principle and potential magnitude of potential benefit, but they still represent in silico results that require experimental farm-specific recalibration and validation using local data on initial infestation, temperature regimes, and mite strains to generate tailored and reliable management schedules, in addition to the extension of toxicological studies on the use of repeated low doses in poultry hens [26].

### Model generality: A flexible framework for parasite-acaricide systems

A key feature of our modelling framework is its inherent adaptability beyond the specific case of *Dermanyssus gallinae* and Fluralaner. While the current parameterisation focuses on PRM and Fluranaler Exzolt®) due to data availability and regulatory relevance, the model’s cohort-structured architecture, temperature-dependent development functions, and economic optimisation logic are deliberately designed as modular components that can be calibrated for other ectoparasites and acaricidal compounds approved for use in laying hens. While this study represents a significant advancement in modelling *D. gallinae* population dynamics in the absence or presence of acaricidal treatment, our model should be regarded as an initial experimental prototype whose reliability requires further validation under field conditions. Furthermore, the model could be improved and optimised by addressing the following aspects:

- Constant environmental assumptions: The model currently assumes constant temperature conditions, justified by the controlled environment of modern poultry houses. However, incorporating dynamic temperature and humidity variables would enhance predictive accuracy across diverse farming systems and geographic regions. Future iterations should integrate the temperature-dependent development functions established by [21] and [10] as continuous inputs rather than static parameters [33].
- Parameter uncertainty: Biological parameters (e.g., development and mortality sub-models) are basically derived from [10,19,21], and [22].Parameter uncertainty and its propagation through the model should be carefully assessed, for instance, through approaches such as Bayesian calibration or ensemble simulations [34]. The recent establishment of stable laboratory PRM colonies [35] provides opportunities for controlled experiments to refine parameter estimates.
- Threshold sensitivity: The optimal threshold $\sigma^{*}\approx 146\;430$ mites per laying hen is sensitive to the assumed cost parameters and production loss functions. Sensitivity analyses, varying these parameters within plausible ranges (e.g., across different production systems, egg prices, and labour costs), would strengthen the robustness of the economic recommendations [36]. Furthermore, the biological plausibility of maintaining populations at this level requires field validation. Does this threshold inadvertently select for faster-reproducing mites through density-dependent mechanisms?

## Conclusion

This cohort-based model provides a significant leap forward in the strategic management of *Dermanyssus gallinae*. The model’s validation against both historical European data and preliminary Tunisian field observations supports its broader applicability across geographic regions and production systems. The integration of detailed mite population dynamics with acaricide pharmacokinetics and an economic optimisation framework–-using Fluralaner as a case study–-opens the way to applying our model through a paradigm shift from eradication to sustainable, threshold-based control. This approach aligns with core IPM principles, preserving acaricide efficacy while minimizing economic and environmental burdens. Importantly, the model’s modular architecture renders it generically applicable to other ectoparasites and acaricidal compounds approved for use in laying hens, provided appropriate species- and compound-specific parameters are available. This flexibility positions it as a unifying platform for integrated parasite management in poultry production. While the model currently operates under constant environmental parameters and requires farm-specific calibration, it establishes a robust, adaptable in silico framework. Further validation under field conditions, along with future work on model optimisation that integrates dynamic environmental variables, parameter uncertainty, and threshold sensitivity, is expected to enhance its practical utility and resilience. This will contribute to the urgently needed advancement of IPM for this globally significant poultry pest.
