# Supplementary material for: A cohort-based population model for the Poultry Red Mite Dermanyssus gallinae (Acari: Dermanyssidae): Application to explore potential pathways for control strategies using Fluralaner as a case study
Source: PLoS One. 2026 Oct 21;21(10):e0357723. doi: 10.1371/journal.pone.0357723 (PMC13686290; doi:10.1371/journal.pone.0357723)
Supplement: Supplementary file 1 [file pone.0357723.s002.pdf]

Response to reviewers

Manuscript ID: PONE-D-26-21612

Title: Optimizing Fluralaner-Based Management Strategies for the Poultry Red Mite Dermanyssus gallinae (Acari: Dermanyssidae): A Cohort-Based Population Model

Dear Academic Editor,

We sincerely thank you for handling our manuscript and for the valuable comments provided by you and the reviewer. We greatly appreciate the time and effort invested in evaluating our work.

We have carefully considered every comment and have revised the manuscript accordingly. We believe that these revisions have substantially improved the clarity, consistency, and reproducibility of our work.

For ease of review, all modifications are detailed below. Reviewer comments are reproduced in bold, followed by our responses. All changes have been colored in red in the tracked-changes version of the manuscript.

Responses to Editorial Requirements

Editorial Comment 1: Please ensure that your manuscript meets PLOS ONE's style requirements, including file naming and formatting.

Response:

Thank you for this comment.

We carefully revised the manuscript formatting according to the official PLOS ONE guidelines, including file organization, section formatting, figure presentation, and manuscript style.

The manuscript has been carefully revised to comply with the official PLOS ONE formatting guidelines. References to figures, tables, and equations have been standardized throughout the manuscript, and the manuscript has been checked for consistency with the journal template.

Editorial Comment 2: **Please ensure compliance with PLOS ONE code sharing policy.**

Response:

Thank you for this comment.

We acknowledge the PLOS ONE code-sharing policy. The source code underlying the analyses will be deposited in a public repository and made freely available without restrictions upon publication of the manuscript.

Editorial Comment 3: **Please revise the Data Availability Statement.**

Response:

Thank you.

The Data Availability Statement has been revised.

Editorial Comment 4: **Please refer to Figure 3 within the manuscript text.**

Response:

Thank you.

A reference to Figure 3 has now been added in the Results section (Page 8/30, Line 162).

Editorial Comment 5: **Please evaluate reviewer-suggested references if applicable.**

Response:

Thank you but Not Applicable

Editorial Comment 6: **Please review the reference list.**

Response:

Thank you.

The entire bibliography has been carefully reviewed.

Formatting has been corrected according to PLOS ONE style.

No retracted references are cited in the manuscript.

Responses to Reviewer #1

General Comment: This manuscript shows the data for novel control strategy of D. gallinae. However, the following things should be considered for publication.

Response:

We sincerely appreciate the reviewer's positive assessment of the scientific quality of our work and thank them for their valuable suggestions. All comments have been carefully addressed as detailed below.

Comment 1: The authors should keep the consistency of scientific terms through the whole manuscript.

Response:

Thank you.

We carefully reviewed the entire manuscript to ensure consistent terminology throughout.

Specifically:

- "Fluralaner" and "fluralaner" are now used consistently according to journal conventions.
- "Exzolt®" is consistently formatted throughout the manuscript.
- Scientific names such as *Dermanyssus gallinae* are now italicized throughout.
- Units have been added wherever numerical values appeared without units (including the Author Summary).

Comment 2: Line 415: Comparaison → Comparison

Response

Corrected.

Comment 3: Line 498: Integrated Pest Management → IPM

Response

Corrected.

The abbreviation "IPM" is now used consistently after its first definition.

Comment 4: Reference formatting should follow journal guidelines.

Response

Thank you.

The reference list has been completely reformatted according to the official PLOS ONE reference style.

All references were checked for consistency, punctuation, capitalization, and formatting.
