# Supplementary material for: A cohort-based population model for the Poultry Red Mite Dermanyssus gallinae (Acari: Dermanyssidae): Application to explore potential pathways for control strategies using Fluralaner as a case study
Source: PLoS One. 2026 Oct 21;21(10):e0357723. doi: 10.1371/journal.pone.0357723 (PMC13686290; doi:10.1371/journal.pone.0357723)
Supplement: Peer review history

# Peer review: Decision Letter 0

**DOI:** 10.1371/journal.pone.0357723.r001

**Authors:** Mário Ferreira Conceição Santos

15 Jul 2026

for the Poultry Red Mite Dermanyssus gallinae

(Acari: Dermanyssidae): A Cohort-Based Population

Dear Dr. Bettaieb,

Thank you for submitting your manuscript to PLOS ONE. After careful consideration, we feel that it has merit but does not fully meet PLOS ONE’s publication criteria as it currently stands. Therefore, we invite you to submit a revised version of the manuscript that addresses the points raised during the review process.

Please submit your revised manuscript by Aug 29 2026 11:59PM. If you will need more time than this to complete your revisions, please reply to this message or contact the journal office at plosone@plos.org. When you're ready to submit your revision, log on to https://www.editorialmanager.com/pone/ and select the 'Submissions Needing Revision' folder to locate your manuscript file.

- A letter that responds to each point raised by the academic editor and reviewer(s). You should upload this letter as a separate file labeled 'Response to Reviewers'.
- A marked-up copy of your manuscript that highlights changes made to the original version. You should upload this as a separate file labeled 'Revised Manuscript with Track Changes'.
- An unmarked version of your revised paper without tracked changes. You should upload this as a separate file labeled 'Manuscript'.

If you would like to make changes to your financial disclosure, please include your updated statement in your cover letter. Guidelines for resubmitting your figure files are available below the reviewer comments at the end of this letter.

If applicable, we recommend that you deposit your laboratory protocols in protocols.io to enhance the reproducibility of your results. Protocols.io assigns your protocol its own identifier (DOI) so that it can be cited independently in the future. For instructions see: https://journals.plos.org/plosone/s/submission-guidelines#loc-laboratory-protocols. Additionally, PLOS ONE offers an option for publishing peer-reviewed Lab Protocol articles, which describe protocols hosted on protocols.io. Read more information on sharing protocols at https://plos.org/protocols?utm_medium=editorial-email&utm_source=authorletters&utm_campaign=protocols.

As the corresponding author, your ORCID iD is verified in the submission system and will appear in the published article. PLOS supports the use of ORCID, and we encourage all coauthors to register for an ORCID iD and use it as well. Please encourage your coauthors to verify their ORCID iD within the submission system before final acceptance, as unverified ORCID iDs will not appear in the published article. *Only*  the individual author can complete the verification step; PLOS staff *cannot*  verify ORCID iDs on behalf of authors.

We look forward to receiving your revised manuscript.

Kind regards,

Mário Ferreira Conceição Santos

Academic Editor

PLOS One

Journal Requirements:

When submitting your revision, we need you to address these additional requirements.

1.Please ensure that your manuscript meets PLOS ONE's style requirements, including those for file naming. The PLOS ONE style templates can be found at

https://journals.plos.org/plosone/s/file?id=wjVg/PLOSOne_formatting_sample_main_body.pdf and

https://journals.plos.org/plosone/s/file?id=ba62/PLOSOne_formatting_sample_title_authors_affiliations.pdf

2. Please note that PLOS One has specific guidelines on code sharing for submissions in which author-generated code underpins the findings in the manuscript. In these cases, we expect all author-generated code to be made available without restrictions upon publication of the work. Please review our guidelines at https://journals.plos.org/plosone/s/materials-and-software-sharing#loc-sharing-code and ensure that your code is shared in a way that follows best practice and facilitates reproducibility and reuse.

3. When completing the data availability statement of the submission form, you indicated that you will make your data available on acceptance. We strongly recommend all authors decide on a data sharing plan before acceptance, as the process can be lengthy and hold up publication timelines. Please note that, though access restrictions are acceptable now, your entire data will need to be made freely accessible if your manuscript is accepted for publication. This policy applies to all data except where public deposition would breach compliance with the protocol approved by your research ethics board. If you are unable to adhere to our open data policy, please kindly revise your statement to explain your reasoning and we will seek the editor's input on an exemption. Please be assured that, once you have provided your new statement, the assessment of your exemption will not hold up the peer review process.

4. Please ensure that you refer to Figure 3 in your text as, if accepted, production will need this reference to link the reader to the figure.

5. If the reviewer comments include a recommendation to cite specific previously published works, please review and evaluate these publications to determine whether they are relevant and should be cited. There is no requirement to cite these works unless the editor has indicated otherwise.

6. Please review your reference list to ensure that it is complete and correct. If you have cited papers that have been retracted, please include the rationale for doing so in the manuscript text, or remove these references and replace them with relevant current references. Any changes to the reference list should be mentioned in the rebuttal letter that accompanies your revised manuscript. If you need to cite a retracted article, indicate the article’s retracted status in the References list and also include a citation and full reference for the retraction notice.

[Note: HTML markup is below. Please do not edit.]

Reviewers' comments:

Reviewer's Responses to Questions

**Comments to the Author**

1. Is the manuscript technically sound, and do the data support the conclusions?

Reviewer #1: Yes

**********

2. Has the statistical analysis been performed appropriately and rigorously?

Reviewer #1: Yes

**********

3. Have the authors made all data underlying the findings in their manuscript fully available?

The PLOS Data policy

Reviewer #1: Yes

**********

4. Is the manuscript presented in an intelligible fashion and written in standard English?

Reviewer #1: Yes

**********

Reviewer #1: This manuscript shows the data for novel control strategy of D. gallinae. However, the following things should be considered for the publication of this journal.

The authors should keep the consistency of scientific terms through the whole manuscript. ex. Fluralaner or fluralaner; Exzolt or Exzolt (R). And, Genus and species name should be intalicized through the whole manuscript. ex. Dermanyssus gallinae. In addition, the data has a unit. ex. 14.15 in Author Summary.

Line 415 : Comparaison --> Comparison

Line 498 : Integrated Pest Management --> IPM

In reference, the format should be followed as the guide recommended by this journal.

ex. reference 12; the type of characters between references are different.

**********

what does this mean?). If published, this will include your full peer review and any attached files.

If you choose “no”, your identity will remain anonymous but your review may still be made public.

**Do you want your identity to be public for this peer review?**  For information about this choice, including consent withdrawal, please see our Privacy Policy

Reviewer #1: **Yes:**  Yun Sang Cho

**********

[NOTE: If reviewer comments were submitted as an attachment file, they will be attached to this email and accessible via the submission site. Please log into your account, locate the manuscript record, and check for the action link "View Attachments". If this link does not appear, there are no attachment files.]

To ensure your figures meet our technical requirements, please review our figure guidelines: https://journals.plos.org/plosone/s/figures

You may also use PLOS’s free figure tool, NAAS, to help you prepare publication quality figures: https://journals.plos.org/plosone/s/figures#loc-tools-for-figure-preparation.

NAAS will assess whether your figures meet our technical requirements by comparing each figure against our figure specifications.

# Author comment: Author response to Decision Letter 1

**DOI:** 10.1371/journal.pone.0357723.r002

18 Aug 2026

Response to reviewers

Manuscript ID: PONE-D-26-21612

Title: Optimizing Fluralaner-Based Management Strategies for the Poultry Red Mite

Dermanyssus gallinae (Acari: Dermanyssidae): A Cohort-Based Population Model

Dear Academic Editor,

We sincerely thank you for handling our manuscript and for the valuable comments provided

by you and the reviewer. We greatly appreciate the time and effort invested in evaluating our

work.

We have carefully considered every comment and have revised the manuscript accordingly.

We believe that these revisions have substantially improved the clarity, consistency, and

reproducibility of our work.

For ease of review, all modifications are detailed below. Reviewer comments are reproduced

in bold, followed by our responses. All changes have been colored in red in the tracked-changes

version of the manuscript.

Responses to Editorial Requirements

Editorial Comment 1: Please ensure that your manuscript meets PLOS ONE's style

requirements, including file naming and formatting.

Response:

Thank you for this comment.

We carefully revised the manuscript formatting according to the official PLOS ONE

guidelines, including file organization, section formatting, figure presentation, and manuscript

style.

The manuscript has been carefully revised to comply with the official PLOS ONE formatting

guidelines. References to figures, tables, and equations have been standardized throughout the

manuscript, and the manuscript has been checked for consistency with the journal template.

Editorial Comment 2: Please ensure compliance with PLOS ONE code sharing policy.

Response:

Thank you for this comment.

We acknowledge the PLOS ONE code-sharing policy. The source code underlying the analyses

will be deposited in a public repository and made freely available without restrictions upon

publication of the manuscript.

Editorial Comment 3: Please revise the Data Availability Statement.

Response:

Thank you.

The Data Availability Statement has been revised.

Editorial Comment 4: Please refer to Figure 3 within the manuscript text.

Response:

Thank you.

A reference to Figure 3 has now been added in the Results section (Page 8/30, Line 162).

Editorial Comment 5: Please evaluate reviewer-suggested references if applicable.

Response:

Thank you but Not Applicable

Editorial Comment 6: Please review the reference list.

Response:

Thank you.

The entire bibliography has been carefully reviewed.

Formatting has been corrected according to PLOS ONE style.

No retracted references are cited in the manuscript.

Responses to Reviewer #1

General Comment: This manuscript shows the data for novel control strategy of D.

gallinae. However, the following things should be considered for publication.

Response:

We sincerely appreciate the reviewer's positive assessment of the scientific quality of

our work and thank them for their valuable suggestions. All comments have been

carefully addressed as detailed below.

Comment 1: The authors should keep the consistency of scientific terms through the

whole manuscript.

Response:

Thank you.

We carefully reviewed the entire manuscript to ensure consistent terminology throughout.

Specifically:

• "Fluralaner" and "fluralaner" are now used consistently according to journal conventions.

• "Exzolt®" is consistently formatted throughout the manuscript.

• Scientific names such as Dermanyssus gallinae are now italicized throughout.

• Units have been added wherever numerical values appeared without units (including the Author

Summary).

Comment 2: Line 415: Comparaison → Comparison

Response

Corrected.

Comment 3: Line 498: Integrated Pest Management → IPM

Response

Corrected.

The abbreviation "IPM" is now used consistently after its first definition.

Comment 4: Reference formatting should follow journal guidelines.

Response

Thank you.

The reference list has been completely reformatted according to the official PLOS ONE

reference style.

All references were checked for consistency, punctuation, capitalization, and format

Attachment Submitted filename: response to reviewers PONE1.pdf

# Editor report: Decision Letter 1

**DOI:** 10.1371/journal.pone.0357723.r003

**Authors:** Mário Ferreira Conceição Santos

22 Aug 2026

A cohort-based population model for the Poultry Red Mite Dermanyssusgallinae (Acari: Dermanyssidae): Application to explore potential pathways for control strategies using Fluralaner as a case study

PONE-D-26-21612R1

Dear Dr.  Maroua Bettaieb

We’re pleased to inform you that your manuscript has been judged scientifically suitable for publication and will be formally accepted for publication once it meets all outstanding technical requirements.

Within one week, you’ll receive an e-mail detailing the required amendments. When these have been addressed, you’ll receive a formal acceptance letter and your manuscript will be scheduled for publication.

An invoice will be generated when your article is formally accepted. Please note, if your institution has a publishing partnership with PLOS and your article meets the relevant criteria, all or part of your publication costs will be covered. Please make sure your user information is up-to-date by logging into Editorial Manager at Editorial Manager® and clicking the ‘Update My Information' link at the top of the page. For questions related to billing, please contact billing support.

If your institution or institutions have a press office, please notify them about your upcoming paper to help maximize its impact. If they’ll be preparing press materials, please inform our press team as soon as possible -- no later than 48 hours after receiving the formal acceptance. Your manuscript will remain under strict press embargo until 2 pm Eastern Time on the date of publication. For more information, please contact onepress@plos.org.

Kind regards,

Mário Ferreira Conceição Santos

Academic Editor

PLOS One

Additional Editor Comments (optional):

Reviewers' comments:

# Editor report: Acceptance letter

**DOI:** 10.1371/journal.pone.0357723.r004

**Authors:** Mário Ferreira Conceição Santos

PONE-D-26-21612R1

PLOS One

Dear Dr. Bettaieb,

I'm pleased to inform you that your manuscript has been deemed suitable for publication in PLOS One. Congratulations! Your manuscript is now being handed over to our production team.

At this stage, our production department will prepare your paper for publication. This includes ensuring the following:

* All references, tables, and figures are properly cited

* All relevant supporting information is included in the manuscript submission,

* There are no issues that prevent the paper from being properly typeset

You will receive further instructions from the production team, including instructions on how to review your proof when it is ready. Please keep in mind that we are working through a large volume of accepted articles, so please give us a few days to review your paper and let you know the next and final steps.

Lastly, if your institution or institutions have a press office, please let them know about your upcoming paper now to help maximize its impact. If they'll be preparing press materials, please inform our press team within the next 48 hours. Your manuscript will remain under strict press embargo until 2 pm Eastern Time on the date of publication. For more information, please contact onepress@plos.org.

You will receive an invoice from PLOS for your publication fee after your manuscript has reached the completed accept phase. If you receive an email requesting payment before acceptance or for any other service, this may be a phishing scheme. Learn how to identify phishing emails and protect your accounts at https://explore.plos.org/phishing.

If we can help with anything else, please email us at customercare@plos.org.

Thank you for submitting your work to PLOS One and supporting open access.

Kind regards,

PLOS One Editorial Office Staff

on behalf of

Dr. Mário Ferreira Conceição Santos

Academic Editor

PLOS One
